# Sox6 and ALDH1A1 Truncation by Asparagine Endopeptidase Defines Selective Neuronal Vulnerability in Parkinson's Disease

**DOI:** 10.1002/advs.202409477

**Published:** 2024-11-21

**Authors:** Shuke Nie, Bowei Li, Mengmeng Wang, Zijun Chen, Jiayan Ren, Zixuan Li, Xinli Xu, Zhengjiang Qian, Zhongyun Xie, Jianxin Han, Zhentao Zhang, Zhaohui Zhang, Yingjie Zhu, Zuxin Chen, Xifei Yang, Keqiang Ye

**Affiliations:** ^1^ Department of Neurology Renmin Hospital of Wuhan University Wuhan Hubei 430060 China; ^2^ Brain Cognition and Brain Disease Institute (BCBDI), Shenzhen Institute of Advanced Technology (SIAT) Chinese Academy of Sciences Shenzhen Guangdong 518055 China; ^3^ Shenzhen Institute of Advanced Technology University of Chinese Academy of Science Shenzhen Guangdong 518055 China; ^4^ Shenzhen Key Laboratory of Drug Addiction Shenzhen Neher Neural Plasticity Laboratory BCBDI SIAT Chinese Academy of Sciences Shenzhen‐Hong Kong Institute of Brain Science‐Shenzhen Fundamental Research Institutions Shenzhen 518055 China; ^5^ Guangdong Provincial Key Laboratory of Brain Connectome and Behavior CAS Key Laboratory of Brain Connectome and Manipulation BCBDI SIAT Chinese Academy of Sciences Shenzhen 518055 China; ^6^ Key Laboratory of Modern Toxicology of Shenzhen Shenzhen Medical Key Discipline of Health Toxicology (2020‐2024) Shenzhen Center for Disease Control and Prevention Shenzhen Guangdong 518055 China; ^7^ Faculty of Life and Health Sciences Shenzhen University of Advanced Technology (SUAT) Shenzhen Guangdong 518107 China

**Keywords:** ALDH1A1, asparagine endopeptidase, parkinson's disease, Sox6, vulnerability

## Abstract

Dopaminergic neurons in the substantia nigra pars compacta (SNpc) demonstrate regionally selective susceptibility in Parkinson's disease (PD) compared to those in the ventral tegmental area (VTA). However, the molecular mechanism for this distinct vulnerability remains unclear. Here, it is shown that Legumain, also known as asparagine endopeptidase (AEP), is activated in a subgroup of SRY‐box transcription factor 6 /Aldehyde dehydrogenase 1 family member A1, (Sox6^+^/ALDH1A1^+^) neurons in the ventral tier of the SNpc and cleaves Sox6 and ALDH1A1, leading to repression of Special AT‐rich sequence binding protein 1 (Satb1) that is a dimeric/tetrameric transcription factor specifically binding to AT‐rich DNA sequences, and toxic dopamine metabolite accumulation. AEP cuts Sox6 and ALDH1A1 in dopaminergic neurons that project to the locus coeruleus (LC), abolishing Sox6's transcriptive and ALDH1A1's enzymatic activities. Co‐expressing AEP‐truncated Sox6 and ALDH1A1 fragments in 3‐month‐old A53T SNCA transgenic mice accelerates dopamine degeneration, whereas expressing AEP‐resistant Sox6 N336A/N446A and ALDH1A1 N220A mutants alleviates rotenone‐induced PD pathologies. Hence, different circuitries and intrinsic properties of dopaminergic neurons in the SNpc and VTA render differential predispositions in PD.

## Graphical abstract

The Gut‐to‐brain propagation of pathologic α‐Syn via vagus nerve‐DMVN‐LC‐SN initiates PD. SNpc DA neurons form stronger connections with the LC than the VTA. AEP is subsequently activated by α‐Syn fibrils in the SNpc, leading to cleavage of Sox6 and ALDH1A1 in SNpc DA neurons, contributing to the vulnerability of dopaminergic neurons in Parkinson's disease.

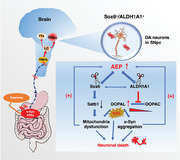

## 1 Introduction

The progressive loss of dopaminergic (DA) neurons in the substantia nigra pars compacta (SNpc) in Parkinson's disease (PD) elicits motor disorders. In contrast, DA neurons in the ventral tegmental area (VTA) are relatively resistant to degeneration. α‐Synuclein (α‐Syn)‐rich intraneuronal protein aggregates named Lewy bodies deposited in remnant SNpc DA neurons are the key feature of PD.^[^ ^1^ ^]^ SNpc DA neurons primarily regulate movement, while VTA DA neurons are involved in reward, emotional behavior, and addiction. Both SNpc and VTA DA neurons produce, store, and release the same neurotransmitter and are located close to each other in the midbrain, with overlapping projections in various brain regions. However, they show different susceptibilities to degeneration in PD. The differential vulnerability to degeneration can be reproduced with the neurotoxin 1‐methyl‐4‐phenyl‐1,2,3,6‐tetrahydropyridine (MPTP) or rotenone in various animal models of PD.^[^ ^2^ ^]^

Kamath et al. developed a molecular classification of human SNpc DA neurons and mapped their spatial distribution within the SNpc. They identified a distinct subpopulation of DA neurons characterized by SRY‐box transcription factor 6 (Sox6) and angiotensin II receptor type 1 (AGTR1), which shows heightened susceptibility to degeneration in PD.^[^ ^3^ ^]^ Notably, the deletion of Sox6 led to a decrease in SNpc markers and an increase in markers commonly linked to the ventral tegmental area (VTA). This loss of Sox6 also affected striatal connections and dopamine levels, with Sox6 expression significantly reduced in PD patients.^[^ ^4^ ^]^ Additionally, aldehyde dehydrogenase 1 (ALDH1A1) is expressed in a subset of DA neurons, predominantly located in the ventrolateral SNpc.^[^ ^5^ ^]^ ALDH1A1 is part of an enzyme family responsible for converting aldehydes into carboxylic acids. ALDH1A1 oxidizes 3,4‐dihydroxyphenylacetaldehyde (DOPAL), a reactive metabolite of dopamine, to detoxify it.^[^ ^6^ ^]^ Inhibition of ALDH1A1 and accumulation of DOPAL contribute to PD pathogenesis.^[^ ^7^ ^]^ Dopaminergic (DA) neurons in the ventral SNpc that express ALDH1A1 are more resistant to degeneration and exhibit fewer α‐Syn aggregates compared to ALDH1A1‐negative DA neurons in the SNpc. Sox6 expression distinguishes ventrally and dorsally biased DA neuron populations in the SNpc. The Sox6^+^ population in the ventral SNpc includes an ALDH1A1^+^ subset and is enriched in gene pathways that exacerbate vulnerability. Sox6^−^ neurons project to the medial, ventral, and caudal striatum and respond to rewards.^[^ ^8^ ^]^

Special AT‐rich sequence binding protein 1 (Satb1) plays a vital role in regulating spatial genome organization, effectively linking higher‐order chromatin architecture with gene regulation.^[^ ^9^ ^]^ Satb1 is a transcriptional regulator identified as a risk factor for PD.^[^ ^10^ ^]^ It controls mitochondrial function and the production of reactive oxygen species (ROS).^[^ ^11^ ^]^ Dysregulation of the Satb1‐MIR22‐GBA pathway, seen in both PD patients and normal aging, contributes to lysosomal and mitochondrial dysfunction.^[^ ^12^ ^]^ Satb1 activity is diminished in the vulnerable brain regions of PD patients.^[^ ^13^ ^]^ As a widely expressed chromatin organizer, Satb1 creates cell type‐specific transcriptional patterns and mediates the response of midbrain DA neurons to toxic stress. The loss of Satb1 triggers a senescence phenotype in human embryonic stem cell (hESC)‐derived DA neurons, while its deletion in these neurons enhances cellular senescence.^[^ ^14^ ^]^

Legumain (LGMN), also known as asparagine endopeptidase (AEP), a cysteine protease that cleaves substrates after asparagine (N), is gradually escalated in the brain with aging and simultaneously cleaves APP N585 to promote Aβ production^[^ ^15^ ^]^ and Tau N368 to facilitate its aggregation,^[^ ^16^ ^]^ triggering Alzheimer's disease (AD) pathologies. Moreover, AEP is progressively activated in PD patient brains in an age‐dependent manner. It cuts α‐Syn N103 and accelerates its fibrillization in PD.^[^ ^17^ ^]^ Remarkably, α‐Syn N103 stimulates monoamine oxidase (MAO) enzymatic activity, leading to DOPAL escalation and AEP augmentation in PD.^[^ ^18^ ^]^ It also fragments UNC5C, a netrin‐1 receptor, at the N467 and N547 residues, facilitating both AD and PD onset.^[^ ^19^ ^]^ DA neurons expressing Sox6 and ALDH1A1,^[^ ^4^, ^20^ ^]^ located in the ventral SNpc, are selectively vulnerable in PD. Moreover, the Sox6^+^ and ALDH1A1^+^ populations exist in the human ventral SNpc and are selectively diminished in postmortem PD brains.^[^ ^8^ ^]^ In the current work, we show that AEP is activated in midbrain DA neurons by α‐Syn fibrils in the SNpc that circuit to the locus coeruleus (LC) via axon projection; in contrast, there is much less neural circuitry between the VTA and LC or dorsal motor vagus nerve (DMVN) in the brain stem. Active AEP cleaves both Sox6 and ALDH1A1, abolishing the former's transcriptive activity and blunting the latter's dehydrogenase activity. As a result, AEP‐fragmented Sox6 is unable to regulate Satb1 expression, and truncated ALDH1A1 fails to metabolize DOPAL, a toxic DA intermediate, leading to selective DA neuronal degeneration in the SNpc compared to the VTA.

## 2 Results

### 2.1 α‐Syn Pathology Preferentially Spreads from the LC to the SNpc Versus the VTA

**Figure 1:**
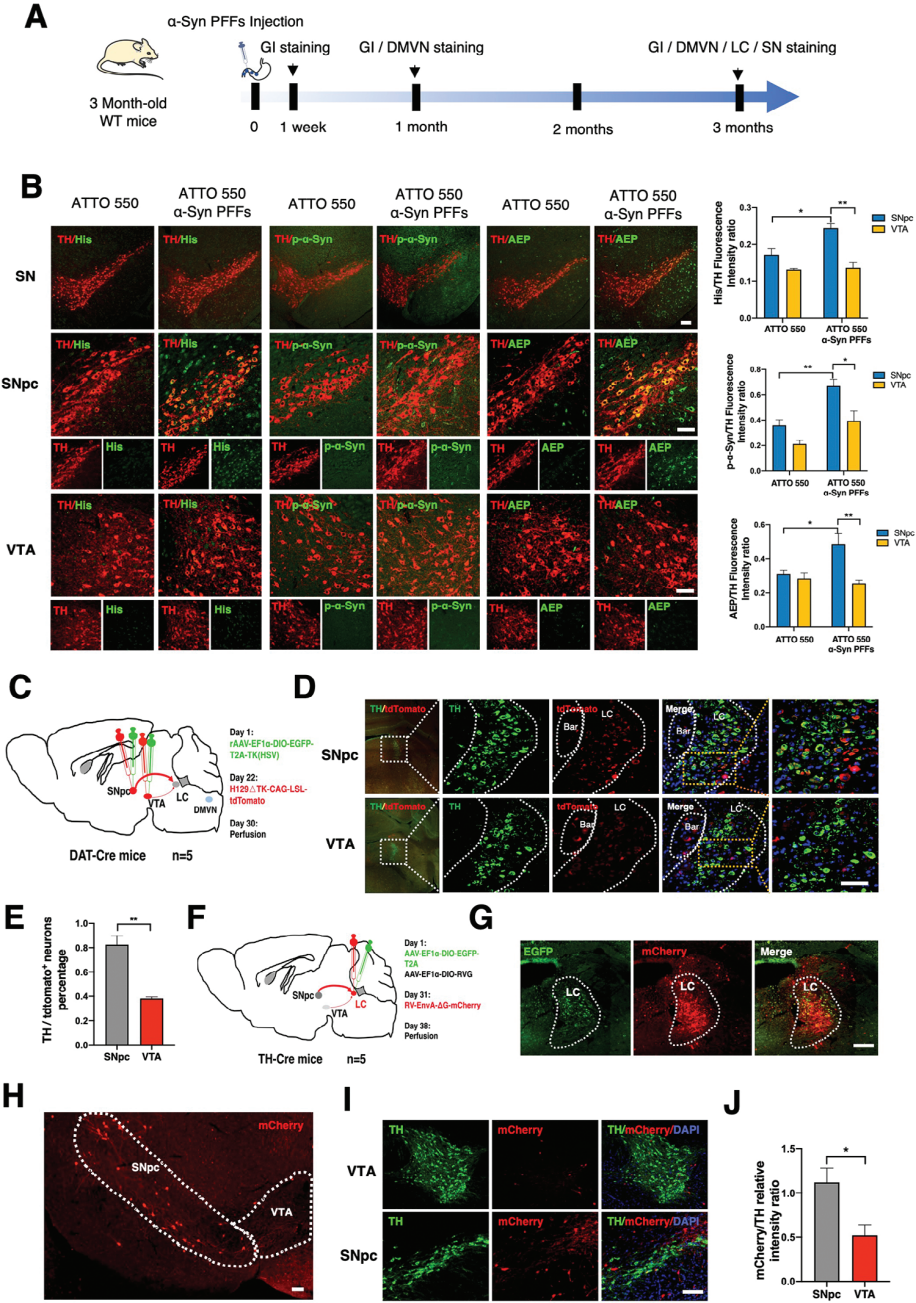
α‐Syn pathology preferentially spreads from the LC to the SNpc versus the VTA. A) Diagram showing the experiment of propagation of pathologic α‐Syn from the gut to the SN. B) Representative immunofluorescence (IF) staining showed that His‐α‐Syn PFFs were distributed more in TH‐positive DA neurons in the SNpc than in the VTA, accompanied by higher p‐S129 α‐Syn and AEP signals. Scale bar = 100 µm in SN, Scale bar = 50 µm in SNpc and VTA. C) Schematic draw showing the anterograde monosynaptic viral tracing strategy to investigate the direct projections from SNpc or VTA dopamine neurons to LC with DAT‐Cre mice (n = 5). D) Representative images showing the anterogradely labeled cells (tdTomato positive) in the LC area from infection of SNpc or VTA dopamine neurons with H129ΔTK‐tdtomato virus. E) Quantification of the percentage of TH positive cells infected by the H129ΔTK‐tdtomato virus in the LC. F) Schematic draw showing the retrograde monosynaptic viral tracing strategy to confirm the direct projection from the SNpc/VTA to the LC TH‐positive neurons with TH‐Cre mice (n = 5). G) Representative image showing the starter cells (EGFP and mCherry double positive) in LC. Scale bar = 25 µm. H,I) Representative images showing the distribution of retrogradely labeled cells (mCherry positive) in the SNpc and VTA. Scale bar = 100 µm in H, Scale bar = 50 µm in I. J) Quantification of mCherry/TH‐positive neurons in the SNpc and VTA. The result suggests that, compared with VTA dopamine neurons, SNpc dopamine neurons have stronger direct connections with the LC TH‐positive neurons. All data are presented as the mean ± SEM from 3 to 6 independent experiments. *p < 0.05; **p < 0.01.

DA neurons in the SNpc are highly vulnerable in PD. We have previously reported that AEP mediates α‐Syn PFFs (preformed fibrils) propagation from the gut to the brain.^[^ ^21^ ^]^ To delineate the underlying mechanisms accounting for the distinct vulnerability, we injected ATTO 550‐conjugated His‐α‐Syn PFFs into the wall of duodenum and stomach in wild‐type mice, the schematic diagram and injection images are shown (Figure S1D,E, Supporting Information). ATTO 550‐labeled α‐Syn PFFs’ infectious activities were validated in HEK293‐α‐Syn stable cells, and the fibrils were also confirmed by transmission electronic microscopy (TEM) (Figure S1A–C, Supporting Information). In three months, we found that His‐tagged PFFs predominantly spread to DA neurons in the SNpc versus the VTA, associated with robust p‐S129 α‐Syn and AEP signals (**Figure** **1**A,B). ATTO 550‐labeled α‐Syn PFFs primarily infected βIII tubulin‐labeled enteric neurons in the duodenum and stomach (Figure S1F, Supporting Information), which elicited demonstrable p‐S129 α‐Syn signals in the enteric neurons (Figure S1G, Supporting Information). His‐tagged PFFs resided in the enteric neurons in both regions one week after fibril injection, and p‐S129 α‐Syn activities in the peripheral neurons lasted more than 1 month (Figure S1H, Supporting Information). Noticeably, ATTO 550‐PFFs incurred substantial p‐S129 α‐Syn activities in choline acetyltransferase (ChaT) neurons in the DMVN; in contrast, rare p‐S129 α‐Syn signals were detectable in mice 1 month after injection with ATTO control (Figure S2A, Supporting Information). Three months after inoculation of PFFs, p‐S129 α‐Syn immunohistochemistry (IHC) signals were prominently more abundant in the SNpc than in the VTA (Figure S2B, Supporting Information). Given the transsynaptic spreading property of α‐Syn, we hypothesized that different levels of accumulation of α‐Syn in SNpc and VTA could be attributed to their different synaptic connectivity with other brain areas such as DMVN and LC. To test this idea, we mapped the direct projection of SNpc and VTA dopamine neurons with monosynaptic anterograde tracing viral system, and found that SNpc dopamine neurons sent stronger direct projections to LC TH‐positive neurons than VTA dopamine neurons (Figure 1C–E). There was no detectable projection from either the VTA or SNpc to the DMVN (Figure S3A,B, Supporting Information), although they projected to numerous different brain regions, including the accumbens nucleus core (ACBC), periaqueductal gray (PAG) from the SNpc, and habenular nucleus (HBN) and nucleus accumbens (NAC) from the VTA (Figure S3C,D, Supporting Information). Consistently, our monosynaptic retrograde mapping study confirmed a stronger projection from the SNpc to LC than the VTA to LC (Figure 1F–J), which was also validated with ALDH1A1 and Sox6 double staining (Figure S3J, Supporting Information). The direct connection between LC and entorhinal cortex was also detected (Figure S3K, Supporting Information). The major cell type of DMVN (Cholinergic neurons) did not form direct synapses on either the SNpc or VTA neurons, but did make synaptic connections with the LC and LSD neurons (Figure S3E–I, Supporting Information). Together, these findings suggest that α‐Syn pathology is more favorably propagated from the LC to DA neurons in the SNpc than in the VTA.

### 2.2 Active AEP in the SNpc Cleaves both Sox6 and ALDH1A1

**Figure 2:**
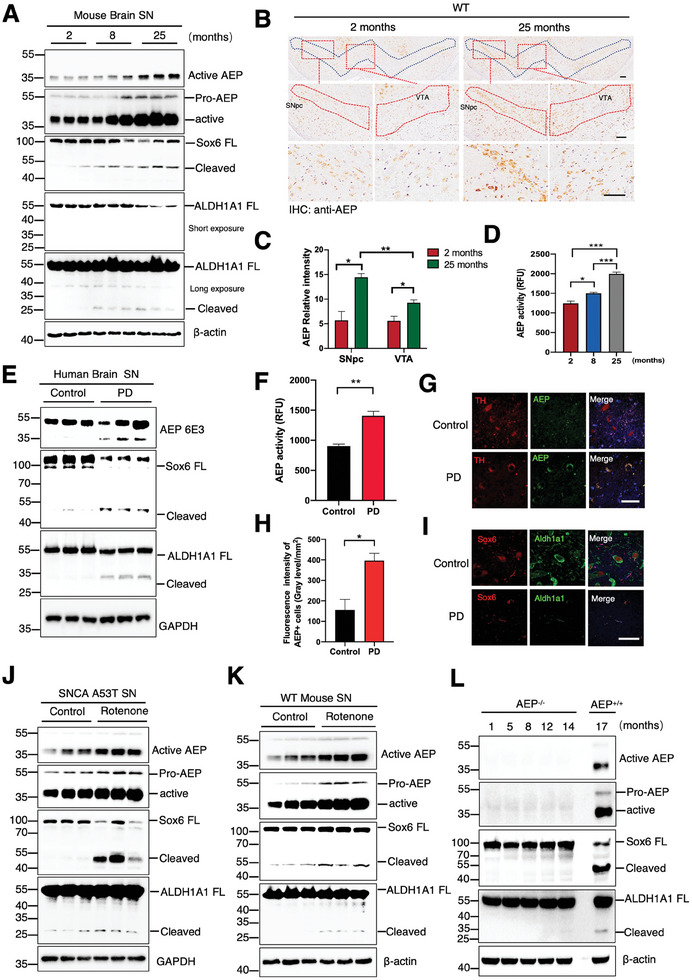
Active AEP in the SNpc cleaves both Sox6 and ALDH1A1. A) Representative immunoblots of the levels of AEP, Sox6, and ALDH1A1 proteins in the SN of aged mice (n = 3 per group). B, C) Representative IHC staining and quantification of the levels of AEP in the SNpc and VTA DA neurons of the mice at different ages. Upper panel: Scale bar = 500 µm, middle panel: Scale bar = 100 µm, bottom panel: Scale bar = 50 µm. *p < 0.05; **p < 0.01. D) AEP enzymatic assay showed that AEP was activated in the brains from different ages. ***p < 0.001. E) Representative immunoblots of the expression of AEP, Sox6, and ALDH1A1 in human SN samples. F) AEP was activated in PD brain tissues. **p < 0.01. G, H) Representative IF staining and quantification of the expression of AEP in PD brains compared to healthy controls. *p < 0.05. I) Representative IF staining showed the expressions of Sox6 and ALDH1A1 were highly reduced in PD brains. Scale bar = 50 µm. J, K) Representative immunoblots showing AEP, Sox6, and ALDH1A1 in the rotenone‐induced PD animal model (n = 3 per group). L) Cleavage of Sox6 and ALDH1A1 was completely abated in AEP^−/−^ mice. (n = 3 per group). All data are presented as the mean ± SEM from 3 to 6 independent experiments. *p < 0.05; **p < 0.01; ***p < 0.001.

Sox6^+^ and ALDH1A1^+^ DA neurons in the SNpc are selectively diminished in postmortem PD brains.^[^ ^8^ ^]^ To test the possibility that active AEP might cut these crucial biomarkers in DA neurons, we conducted cellular experiments and found that rotenone dose‐dependently activated AEP, correlating with gradual escalation of both Sox6 and ALDH1A1 truncation in SH‐SY5Y cells, which were blocked by AEP inhibitor CP#11A (Figure S4A,B, Supporting Information). IF analysis showed that both of them were repressed by rotenone in primary dopaminergic neurons (Figure S4C, Supporting Information). To examine the pathological roles of AEP in the SNpc, we performed immunoblotting (IB) with brain lysates from WT mice of different ages and found that AEP was progressively activated, coupled with gradually truncated Sox6 and ALDH1A1 (**Figure** **2**A,D; Figure S5A, Supporting Information). IHC analysis revealed that AEP was significantly activated at 25 months of age versus 2 months in both the SNpc and VTA regions, with the former much stronger than the latter (Figure 2B,C), which was also confirmed by immunofluorescence (IF) staining (Figure S4D, Supporting Information). Both Sox6 and ALDH1A1 signals were greatly reduced in the SNpc from 25‐month‐old mice compared to 2‐month‐old mice, whereas they remained unchanged in the VTA (Figure S4E–H, Supporting Information). IB and IF staining with PD patient samples indicated that AEP was strongly activated, accompanied by prominent Sox6 and ALDH1A1 fragmentation compared to controls (Figure 2E,F; Figure S5B, Supporting Information). IF study uncovered that AEP was significantly augmented in TH‐positive neurons in PD brains compared with controls (Figure 2G,H). Accordingly, both of Sox6 and ALDH1A1 fluorescent activities were highly reduced in PD brains, associated with significant TH loss (Figure 2I; Figure S5C–F, Supporting Information). IHC and IF studies supported that AEP was strongly activated by rotenone in DA neurons in SNpc versus VTA, fitting with extensive TH loss in the striatum upon rotenone treatment (Figure S6F–H, Supporting Information). In the rotenone‐treated A53T PD mouse model, AEP was robustly activated and correlated with heightened expression of fragmented Sox6 and ALDH1A1, as well as reduced expression of full‐length Sox6 and ALDH1A1, in comparison to WT mice (Figure 2J,K; Figure S6A–E, Supporting Information). These events were completely abated in AEP^−/−^ mice at different age (Figure 2L), supporting that AEP is implicated in shredding both Sox6 and ALDH1A1 in the SN region in PD. Satb1, a DNA‐binding protein linked to PD, functions as an endogenous neuroprotective factor that may have evolved to protect SNpc DA neurons. It is essential for maintaining the dopaminergic phenotype, and disruption of its activity leads to DA neuron degeneration.^[^ ^13^ ^]^ Satb1 and Sox6 are co‐expressed in DA neurons in the SNpc.^[^ ^22^ ^]^ Interestingly, GEO DataSet re‐analysis showed that *satb1* mRNA was significantly reduced in both the lateral and medial SN regions in PD, fitting with Sox6 mRNA levels (Figure S6I–K, Supporting Information).^[^ ^23^ ^]^ Consequently, deletion of Sox6 in SH‐SY5Y cells evidently repressed both Satb1 and cytochrome c oxidase IV (COX IV) expression (Figure S6L–N, Supporting Information). Hence, Sox6 may regulate Satb1 expression in neurons, in alignment with previous report.^[^ ^8^ ^]^

### 2.3 AEP Proteolytically Cleaves Sox6 and ALDH1A1, Triggering Mitochondrial Dysfunction and DA Neuronal Death

**Figure 3:**
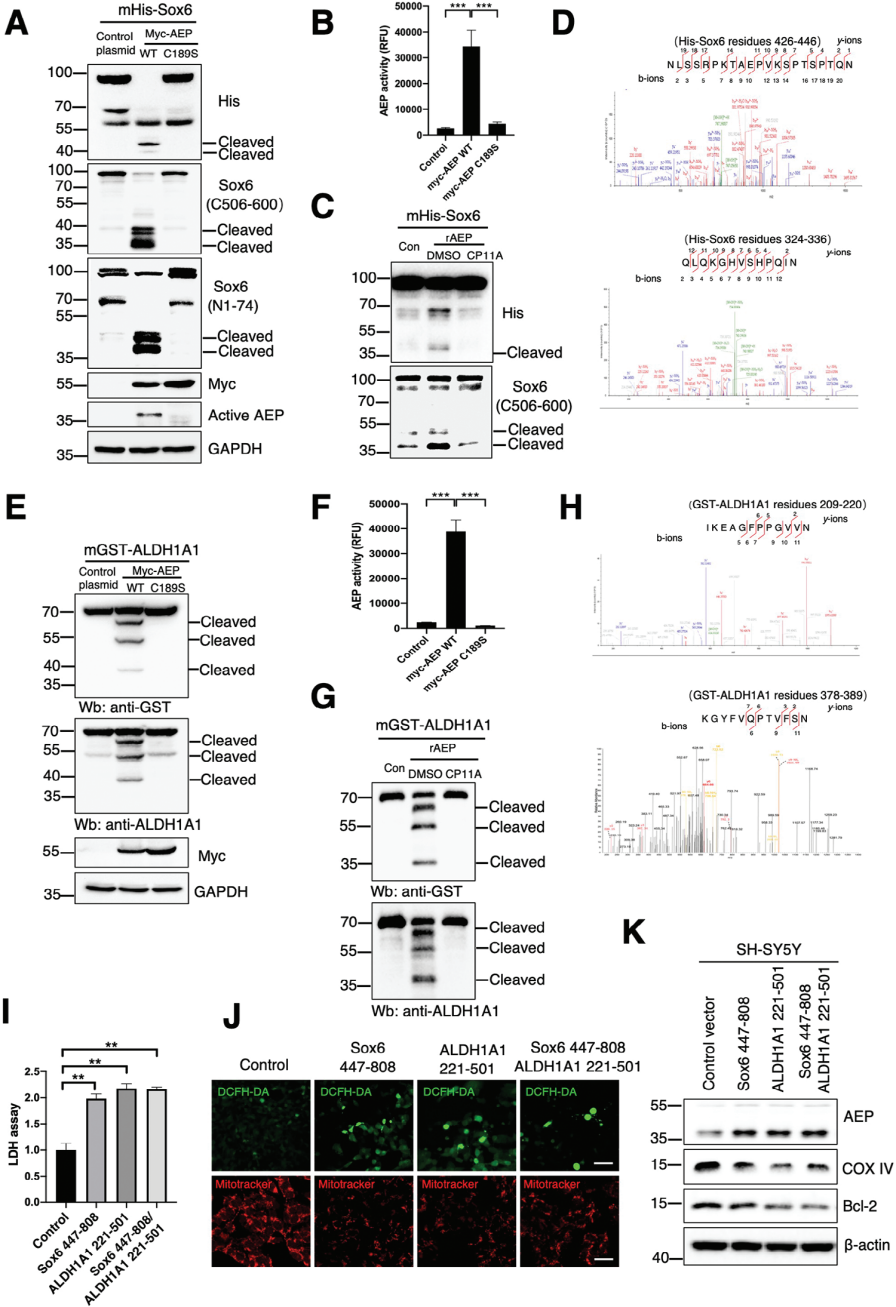
AEP proteolytically cleaves Sox6 and ALDH1A1, triggering mitochondrial dysfunction and dopaminergic neuronal death. A) Co‐transfection of Myc‐AEP or the C189S mutant with His‐Sox6 into HEK293 cells showed that His‐Sox6 was fragmented by AEP, which was blocked in Myc‐AEP C189S cells. B) The AEP enzymatic assay showed that AEP activity was diminished by the C189S mutant of AEP. ***p < 0.001.C) Sox6 fragmentation was blocked by the AEP inhibitor CP#11A in cells. D) LC/MS/MS analysis identified N336 and N446 as the major cutting sites on Sox6. E) Co‐transfection of Myc‐AEP or the C189S mutant with GST‐ALDH1A1 into HEK293 cells showed that ALDH1A1 was fragmented by AEP, which was blocked in C189S cells. F) Enzymatic assays showed that AEP activity was diminished by the C189S mutant of AEP. ***p < 0.001.G) ALDH1A1 fragmentation was blocked by the AEP inhibitor CP#11A in cells. H) LC/MS/MS analysis identified that N220 and N389 were the major cutting sites on ALDH1A1. I) LDH assays showed that Sox6 447–808 and the ALDH1A1 221–501 fragment induced cell death. J) Representative images showing Sox6 447–808 and ALDH1A1 221–501 fragment‐triggered mitochondrial dysfunction and ROS elevation in SH‐SY5Y cells. Scale bar = 100 µm. K) Representative immunoblots showing AEP, COX IV, and Bcl‐2 in Sox6 447–808 and ALDH1A1 221–501 fragment‐treated cells. All data are presented as the mean ± SEM from 3 independent experiments. ** *p* < 0.01; ***p < 0.001.

To map the cutting sites on Sox6 by AEP, we performed an in vitro cleavage assay with recombinant AEP, and found that Sox6 was truncated into multiple fragments in a time‐dependent manner, and these results were demonstrated by different antibodies (Figure S7A,B, Supporting Information). Co‐transfection of Myc‐AEP or enzymatic‐dead C189S mutant with His‐Sox6 into HEK293 cells showed that His‐Sox6 was fragmented by AEP, which was blocked in C189S cells, and fragmentation was also blocked by AEP inhibitor CP11A (**Figure** **3**A–C). LC/MS/MS analysis identified that N336 and N446 were the major cutting sites on Sox6 (Figure 3D). The N336A or N446A mutant blunted Sox6 fragmentation, and N336/446A substantially abrogated Sox6 cleavage (Figure S7C, Supporting Information). Subcellular fractionation revealed that full‐length Sox6 (FL) mainly resided in the nucleus, while N336 or N446 fragments augmented their cytoplasmic distribution (Figure S7D, Supporting Information). Interestingly, Sox6 with N to A mutants were predominantly allocated to the nucleus (Figure S7E, Supporting Information). Consistently, IF analysis indicated that Sox6 primarily resided in the nucleus of DA neurons in 2‐month‐old mice, while some were redistributed in the cytoplasm in 25‐month‐old mice (Figure S7F, Supporting Information). To explore the effects of Sox6 fragmentation on the roles of Satb1 in the mitochondria, we transfected SH‐SY5Y cells with FL Sox6 and AEP‐generated Sox6 fragments. Sox6 447–808 fragment exhibited much stronger repressive effect on Satb1 and COX IV expression than 337–808 truncate, which was further augmented in the presence of rotenone (Figure S7G,H, Supporting Information).

In vitro cleavage assays with GST‐ALDH1A1 showed that AEP strongly cleaved it into fragments (Figure S8A,B, Supporting Information). Knockout of AEP from the brain abolished its fragmentation (Figure S8C,D, Supporting Information). Co‐transfection of GST‐ALDH1A1 and AEP into HEK293 cells yielded prominent fragmentation, which was abolished by AEP C189S mutation. Interestingly, ALDH1A1 truncation was diminished when the cells were exposed to the specific AEP inhibitor CP#11A (Figure 3E–G).^[^ ^24^ ^]^ LC/MS/MS indicated that N220 and N389 residues were the primary cutting sites on ALDH1A1 (Figure 3H). The mutation assay supported that N220 was the predominant cutting site on ALDH1A1 (Figure S8E, Supporting Information). Enzymatic assays showed that cleavage at N220 disrupted ALDH1A1 activity (Figure S8F, Supporting Information). Physiologically, ALDH1A1 detoxifies DOPAL, a dopamine metabolite that is toxic by MAO‐B, into 3,4‐dihydroxyphenylacetic acid (DOPAC). Consequently, fragmentation of ALDH1A1 enhanced SH‐SY5Y cell death, which was further augmented by DOPAL. Notably, AEP‐resistant N220A mutant significantly suppressed DOPAL‐induced cell death (Figure S8G,H,I, Supporting Information). In primary neuronal cultures, α‐Syn PFFs elicited neuronal cell death, which was further exacerbated by DOPAL (Figure S8J,K, Supporting Information). AEP cleavage of Sox6 or ALDH1A1 crippled their physiological roles and induced mitochondrial dysfunction and cell death. As a result, Sox6 447–808 or ALDH1A1 221–501 fragment alone triggered mitochondrial membrane potential impairment and ROS elevation in SH‐SY5Y cells. The combination of Sox6 447–808 and ALDH1A1 221–501 demonstrated an deleterious effect on Bcl‐2, an anti‐apoptotic biomarker, and DCFH‐DA, a mitochondrial membrane potential biomarker (Figure 3I–K; Figure S8L,M, Supporting Information). Accordingly, AEP was highly activated, and COX IV was reduced, in accordance with decreased Bcl‐2, a marker for cell survival (Figure 3I–K).

### 2.4 AEP Cleaves Sox6 and ALDH1A1 in PD, Diminishing Satb1 and Escalating DOPAL in the SNpc

**Figure 4:**
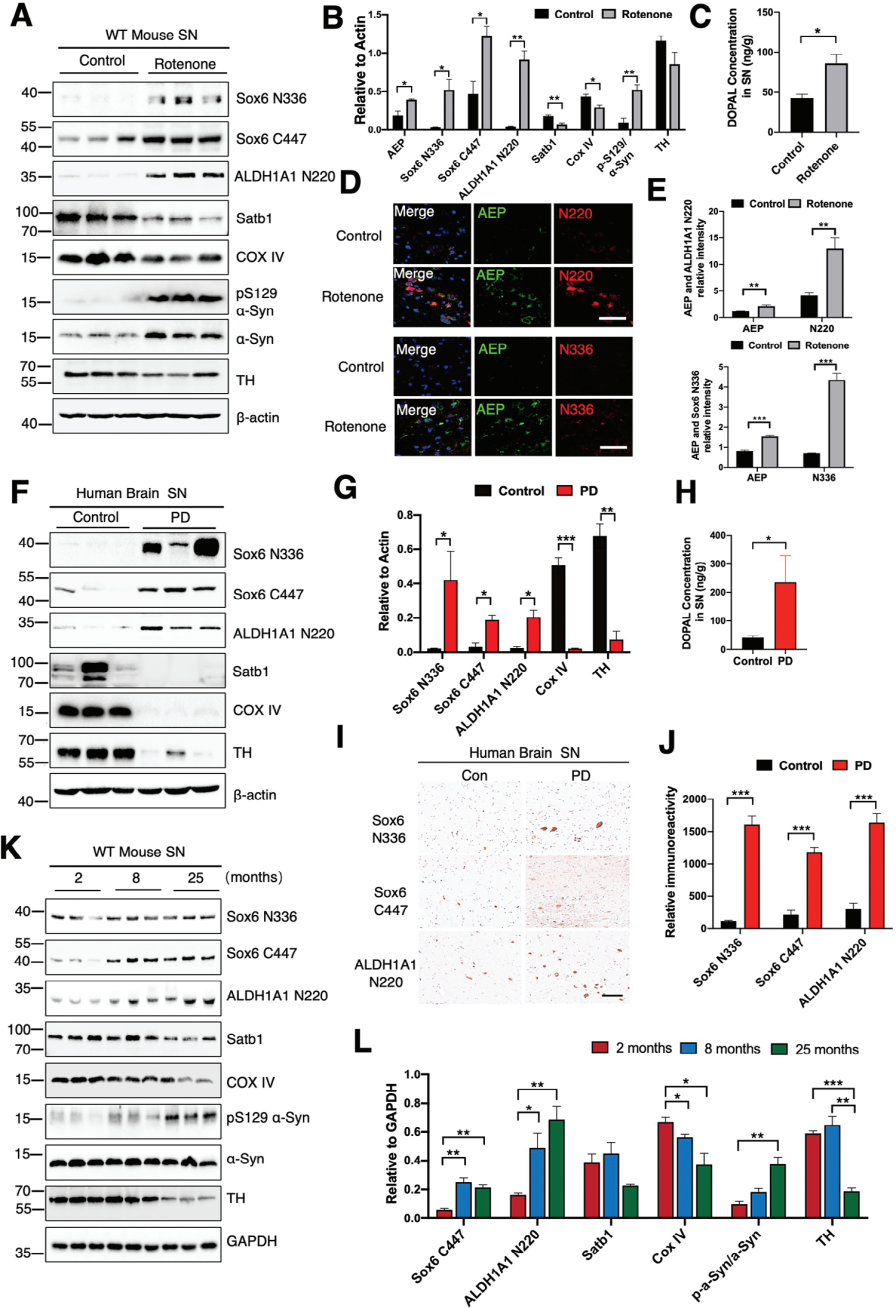
AEP cleaves Sox6 and ALDH1A1 in PD, diminishing Satb1 and escalating DOPAL in the SNpc. A, B) Representative immunoblots and quantification of the levels of Sox6 N336/N446, ALDH1A1 N220, Satb1, COXIV, pS129 α‐Syn/α‐Syn and TH in the SN of the rotenone‐induced PD animal model (n = 3 per group). *p < 0.05; **p < 0.01. C) DOPAL concentrations increased in the SN of the rotenone‐induced PD model. D, E) IF staining and quantification showed that Sox6 and ALDH1A1 were highly cleaved by active AEP in the SNpc. Scale bar = 20 µm. F) Robust Sox6 and ALDH1A1 cleavage by AEP, accompanied by complete eradication of Satb1 and COX IV and diminished TH levels in SN tissues of PD patient. G) Quantification of the levels of the above proteins in PD patient SN tissues. *p < 0.05; **p < 0.01; ***p < 0.001. H) DOPAL levels were significantly increased in PD SN tissues, consistent with ALDH1A1 proteolytic fragmentation. *p < 0.05. I, J) Representative IHC staining and quantification of Sox6 N336, Sox6 C447, and ALDH1A1 N220 fragmentation in PD patient SN slices. Scale bar = 20 µm. K) Immunoblotting showing Sox6 N336, Sox6 C447, ALDH1A1 N220 fragmentation escalation, Satb1, COXIV, pS129 α‐Syn/α‐Syn, and TH levels in the mouse SN from 2 to 8 to 25 months of age (n = 3 per group). L) Quantification of the levels of the above proteins in the SN of different mice. (All data are presented as the mean ± SEM from 3 to 6 independent experiments. *p < 0.05; **p < 0.01; ***p < 0.001.

To interrogate the pathological roles of Sox6 and ALDH1A1 cleavage by AEP, we treated 6‐month‐old C57BL/6J mice with low dose of rotenone (1 mg kg^−1^, i.p.),^[^ ^25^ ^]^ which was previously shown to exhibit most PD pathologies and motor disorders. Two months later, IB analysis showed that rotenone stimulated robust AEP activation in the SN tissues, coupled with prominent Sox6 N336 and N446 and ALDH1A1 N220 fragmentation (**Figure** **4**A,B). Satb1 is more abundantly expressed in DA neurons in the SNpc than the VTA, and its reduction elicits DA neurodegeneration.^[^ ^13^ ^]^ Satb1 is vital for maintaining mitochondrial mass and functions.^[^ ^14^ ^]^ Sox6 mediated Satb1 expression (Figure S6I–K, Supporting Information). In agreement with loss of function of Sox6, both Satb1 and mitochondrial complex IV (COX IV) components were conspicuously repressed by rotenone. Correspondingly, α‐Syn was greatly augmented and associated with noticeable p‐S129 activities (Figure 4A,B). Remarkably, DOPAL levels were significantly escalated upon rotenone treatment (Figure 4C), consistent with ALDH1A1 proteolytic fragmentation. IF showed that Sox6 and ALDH1A1 were highly cleaved by active AEP in DA neurons in the SNpc (Figure 4D,E). Consistent with previous findings, dopaminergic neurodegeneration in α‐Syn transgenic mice primarily affects a dorsomedial ALDH1A1‐positive subpopulation, which is also more susceptible to cytotoxic α‐Syn aggregation.^[^ ^5a^ ^]^ In PD patient SN tissues, we observed more extreme Sox6 and ALDH1A1 cleavage by AEP, accompanied by complete eradication of Satb1 and COX IV. Accordingly, TH was substantially diminished, and DOPAL was strongly increased in PD patients compared to controls (Figure 4F–H). These effects were further corroborated by IHC staining of PD brain tissues (Figure 4I,J). We basically made similar observations in gradually aged mice with these biological events progressively escalating from 2 to 8 and then to 25 months of age (Figure 4K,L). We observed a significant decrease in the levels of TH and Satb1 in the midbrain of 25‐month‐old mice, indicating that changes in TH and Satb1 only occur when there is a certain degree of loss in dopaminergic neurons. AEP in the substantia nigra of 8‐month‐old WT mice was significantly activated (Figure 2D), suggesting that the early activation of AEP in shredding Sox6 and ALDH1A1 is an early pathological feature of PD. Therefore, AEP cleaves both Sox6 and ALDH1A1, reducing Satb1 levels and increasing DOPAL concentrations in PD.

### 2.5 Cleavage of Sox6 and ALDH1A1 by AEP Triggers DA Neuronal Loss and Motor Disorders

**Figure 5:**
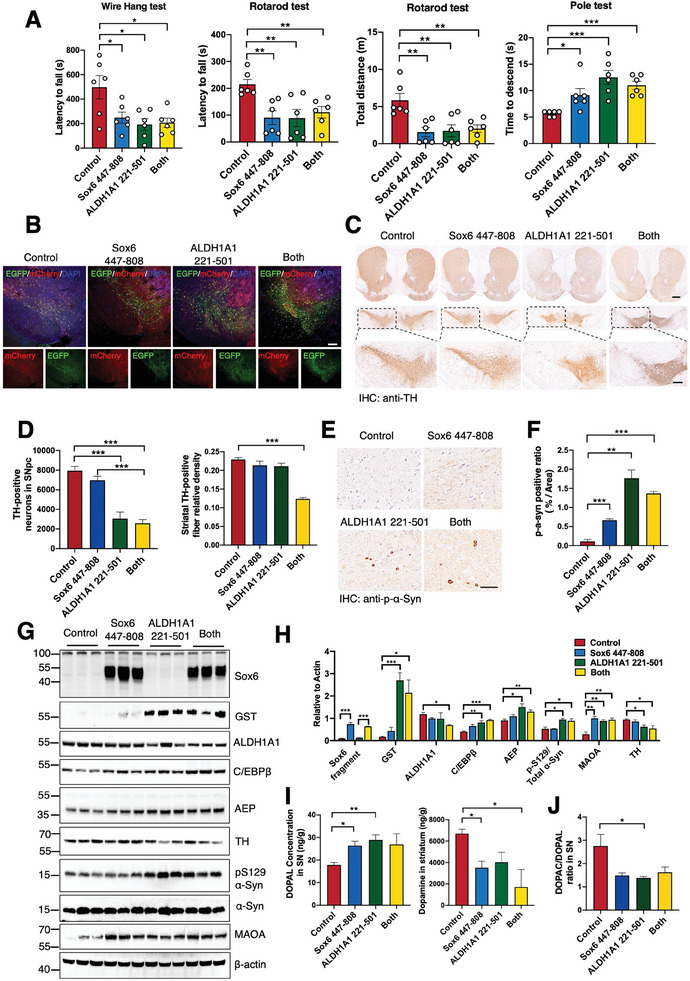
Sox6 and ALDH1A1 fragments cleaved by AEP trigger dopaminergic loss and motor disorders. A) Three months after AAV‐Sox6 447–808 or AAV‐ALDH1A1 221–501 or their combination injection, the wire hang test (left), rotarod test (middle) and pole test (right) were performed in SNCA A53T mice. B) Co‐staining mCherry and EGFP in the SNpc of SNCA A53T mice injected with various viruses. Scale bar = 100 µm. C, D) Representative TH staining and quantification of striatum and SNpc/VTA in the four groups (n = 3 per group). (Scale bars, upper panel 400 µm; lower panel, 100 µm). ***p < 0.001. E, F) Representative pS129 α‐Syn staining and quantification of SNpc DA neurons in SNCA A53T mice at three months after injection of AAV vectors, AAV‐Sox6 447–808, AAV‐ALDH1A1 221–501, and AAV‐Sox6 447–808/AAV‐ALDH1A1 221–501 in the SNpc of A53T mice. Scale bar = 100 µm. G, H) Representative immunoblots and quantifications showed ALDH1A1 221–501 and combination injection increased AEP level, coupled with augmented p‐S129 α‐Syn activities and down‐regulated TH signals. (n = 3 per group). I) Over‐expressions of Sox6 and ALDH1A1 fragments in SNpc resulted in elevated toxic DOPAL level and reduced DA concentration. (n = 3 per group). *p < 0.05; **p < 0.01; ***p < 0.001. J) DOPAC/DOPAL ratios were significantly decreased when ALDH1A1 221–501 was overexpressed. *p < 0.05.

Because Sox6 447–808 fragment exhibits much stronger repressive effects on Satb1 and COX IV expression than 337–808 truncate in the presence of rotenone, we chose to inject AAV virus expressing either EGFP‐Sox6 447–808 and mCherry‐ALDH1A1 221–501 or their combination into the SNpc regions (AP ‐3.2 mm; ML ±1.4 mm; DV ‐4.15 mm) of 3‐month‐old A53T mice, IF co‐staining of EGFP and mCherry in the SNpc area were used to confirm proper viral injections (**Figure** **5**B). Three months after injection, motor functional assays showed that overexpression of Sox6 447–808 or ALDH1A1 221–501 or both in the SN region elicited great motor disorders (Figure 5A). Sox6 447–808 group and both groups exhibited a significant decrease in striatal dopamine levels (Figure 5I), resulting in motor dysfunction in mice. IHC study revealed extensive TH neuronal loss in the SN as compared to VTA regions, associated with pronounced p‐S129 α‐Syn signals (Figure 5C–F). The levels of tyrosine hydroxylase (TH) in the substantia nigra and striatum of Sox6 447–808 group of mice showed no significant changes (Figure 5C,D), suggesting that dopamine synthesis may not be affected. However, there was a significant increase in the levels of the dopamine metabolite DOPAL, indicating accelerated oxidative metabolism of dopamine. Through the assessment of MAO‐A expression levels, we found that Sox6 447–808 significantly enhanced the expression of MAO‐A (Figure 5G). Therefore, the reduction of dopamine levels by Sox6 447–808 mainly due to increased dopamine oxidation into DOPAL without significantly diminishing dopamine synthesis in TH positive neurons. IB demonstrated that these fragments were abundantly expressed. Remarkably, AEP was significantly elevated in ALDH1A1 221–501 or both expressed brains, coupled with augmented p‐S129 α‐Syn activities, which were conversely accompanied with TH signals (Figure 5G,H). CCAAT‐enhancer‐binding protein (C/EBPβ), an inflammation‐regulated transcription factor, acts as a key age‐dependent effector elevating both AEP and inflammatory cytokines expression in mediating pathogenesis in PD mouse models.^[^ ^26^ ^]^ We found the expression levels of C/EBPβ in ALDH1A1 221–501 or both expressed brains robustly increase, consistent with the expression of active AEP. Correspondingly, DOPAL concentrations were highly increased, and DA was inversely reduced in these samples (Figure 5I). As expected, DOPAC/DOPAL ratios were significantly decreased when ALDH1A1 221–501 was overexpressed (Figure 5J).

### 2.6 Sox6 and ALDH1A1 Resistant Mutants Protect Dopaminergic Neuron Loss and Motor Disorder from AEP Cleavage

**Figure 6:**
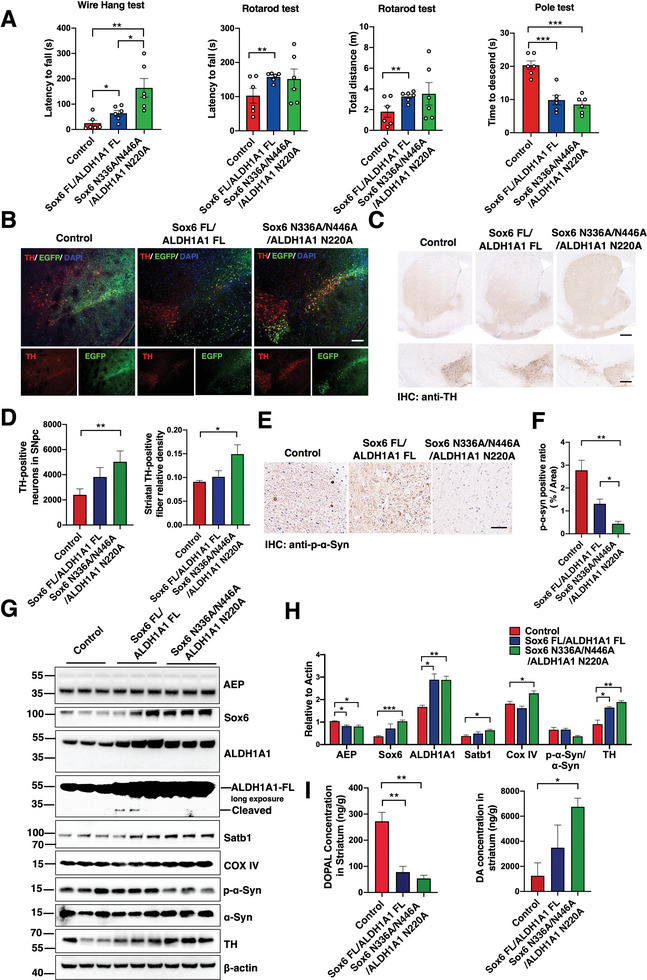
Sox6 and ALDH1A1 resistant mutants protect dopaminergic neuron loss and motor disorder from AEP cleavage. A) Three months after AAV‐Sox6 FL/AAV‐ALDH1A1 FL or AAV‐Sox6 N336A/N446A/AAV‐ALDH1A1 N220A injection and low‐dose rotenone treatment (2.5 mg kg^−1^), the wire hang test (left), rotarod test (middle) and pole test (right) were performed in SNCA A53T mice. B) Co‐staining TH and EGFP in the SNpc of rotenone‐treated SNCA A53T mice injected with various viruses. Scale bar = 100 µm. C, D) Representative IHC TH staining and quantification of the striatum and the SNpc/VTA in the control, AAV‐Sox6 FL/AAV‐ALDH1A1 FL, and AAV‐Sox6 N336A/N446A/ALDH1A1 N220A groups (Scale bars, upper panel 400 µm; lower panel 100 µm). *p < 0.05; **p < 0.01. E, F) Representative pS129 α‐Syn staining of SNpc DA neurons in SNCA A53T mice at three months after injection of AAV vectors, AAV‐Sox6 FL/AAV‐ALDH1A1 FL, and AAV‐Sox6 N336A/N446A/ALDH1A1 N220A in the SNpc of rotenone‐treated A53T mice (n = 3 per group). *p < 0.05; **p < 0.01. Scale bar = 50 µm. G, H) Representative immunoblots and quantifications showed AEP uncleavable mutants on the levels of AEP, Satb1, COX IV, p‐α‐Syn, and TH proteins. (n = 3 per group). *p < 0.05; **p < 0.01; ***p < 0.001.I) Over‐expressions of uncleavable Sox6 and ALDH1A1 proteins in SNpc resulted in decreased toxic DOPAL level and increased DA concentration. All data are presented as the mean ± SEM from 3 to 6 independent experiments. *p < 0.05; **p < 0.01; ***p < 0.001.

Usually, the genetic mutant SNCA A53T transgenic mice only develop a portion of PD pathologies at very old age, and they begin to reveal motor impairments ≈10 months of age.^[^ ^27^ ^]^ In order to present more featured Lewy body‐like pathologies, we employed rotenone in 3‐month‐old SNCA A53T mice reported in previous literature.^[^ ^21^ ^]^ To investigate whether blockade of AEP cleavage alleviates the pathological effects, we injected AAV expressing AEP‐resistant EGFP‐Sox6 N336A/N446A and EGFP‐ALDH1A1 N220A mutants or FL counterparts into the SN regions(AP ‐3.2 mm; ML ±1.4 mm; DV −4.15 mm) in A53T mice, followed by three months rotenone treatment (2.5mg Kg^−1^, i.g.). IF co‐staining of TH and EGFP showed accurate viral injection and expression in mice SN (**Figure** **6**B). Compared with control virus or FL group, AEP uncleavable mutants significantly elevated various motor functions (Figure 6A). In accordance with these effects, IHC staining showed abundant TH signals in AEP‐resistant mutants compared to FL and control groups. As expected, p‐S129 α‐Syn levels were strongly blunted in the mutant groups in comparison to control or FL group (Figure 6C–F). IB analysis indicated that Sox6 and ALDH1A1 FL and their uncleavable counterparts were highly expressed in the brains, and rotenone treatment elicited Sox6 and ALDH1A1 fragmentation, which was abolished in the mutant groups. Complying with these events, Satb1 and COX IV levels were elevated in the mutant group; conversely, p‐S129 α‐Syn was obviously repressed. Consequently, TH levels were significantly escalated in the SN (Figure 6G,H). Accordingly, DOPAL levels were greatly decreased in the mutant group, and DA concentrations were highly enhanced (Figure 6I).

### 2.7 Knockdown of Sox6 and ALDH1A1 Accelerates the PD Pathology in SNCA A53T Mice

**Figure 7:**
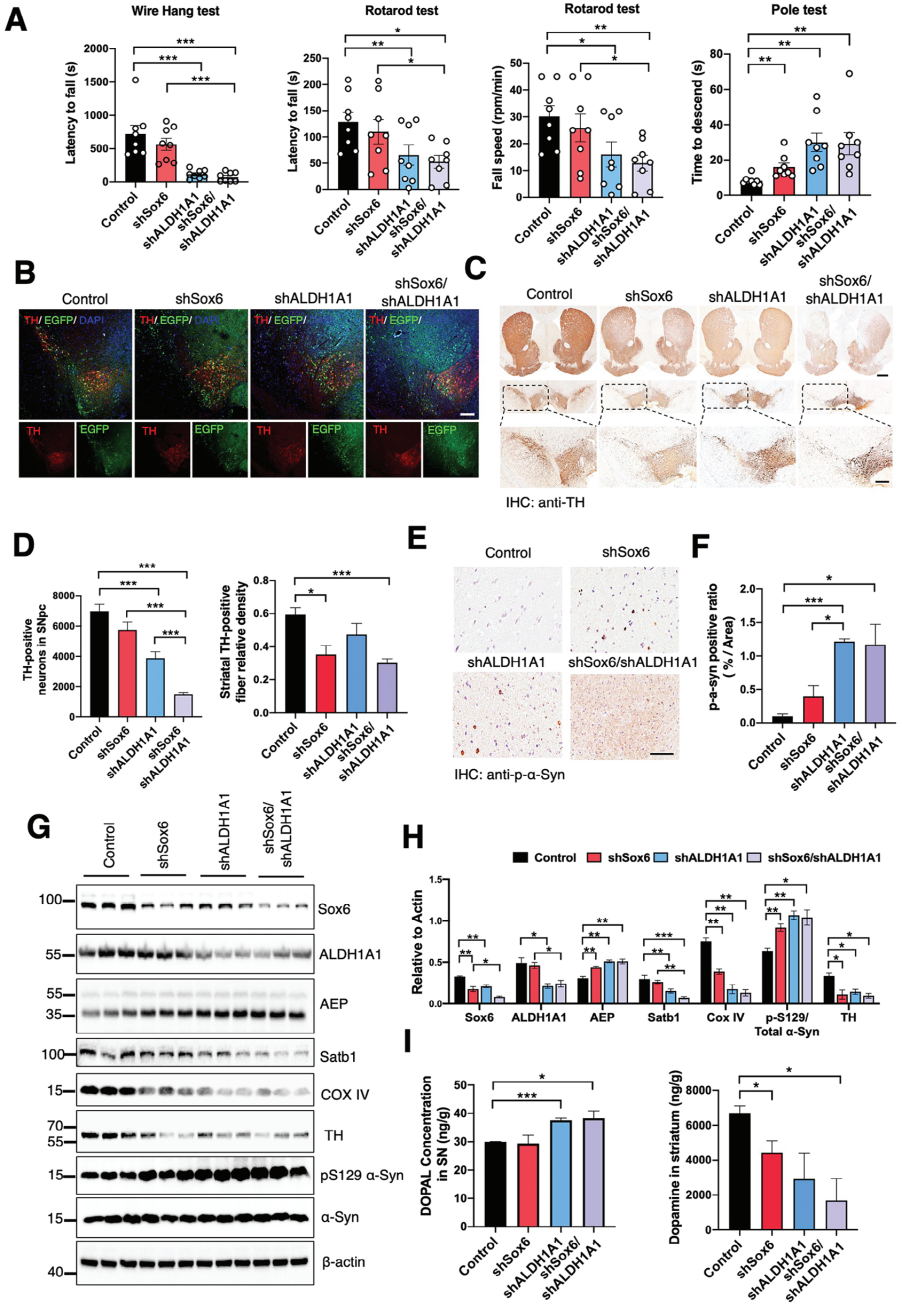
Knockdown Sox6 and ALDH1A1 accelerate the PD pathology in the SNCA A53T mice. A) Three months after AAV‐shSox6 or AAV‐shALDH1A1 or their combination injection, wire hang test (left), rotarod test (middle), and pole test (right) were performed in SNCA A53T mice. B) Co‐staining TH and EGFP in the SNpc of SNCA A53T mice injected with various viruses. Scale bar = 100 µm. C, D) Representative TH staining and quantification of striatum and SNpc/VTA in the four groups (n = 3 per group). (Scale bars, above 400 µm, below 100 µm). * *p* < 0.05; ** *p* < 0.01; ***p < 0.001. E) Representative pS129 α‐Syn staining of SNpc DA neurons of SNCA A53T mice at three months after injection of AAV‐shVectors, AAV‐shSox6, AAV‐shALDH1A1, AAV‐shSox6/AAV‐shALDH1A1 in the SNpc of A53T mice. (Scale bars, 100 µm). F) Quantifications of pS129 α‐Syn staining in SNpc. (n = 3 per group). * *p* < 0.05; ** *p* < 0.01; ***p < 0.001. G, H) Representative immunoblots and quantifications showed knockdown the Sox6 and ALDH1A1 on the levels of AEP, Satb1, COX IV, p‐S129 α‐Syn activities, and TH proteins. (n = 3 per group). I) Knockdown of Sox6 and ALDH1A1 in SNpc resulted in elevated toxic DOPAL level and reduced DA concentration. * *p* < 0.05; ** *p* < 0.01; ***p < 0.001.

To further interrogate the roles of Sox6 and ALDH1A1 in protecting DA neurons, we depleted each of them or in a combination using EGFP‐shSox6 and EGFP‐shALDH1A1 virus in 3‐month‐old SNCA A53T mice. The injection site and virus expression were confirmed using TH/EGFP IF co‐staining (**Figure** **7**B). We found that combined deletion significantly crippled motor functions in the behavior tests than separate deletion, indicating Sox6 and ALDH1A1 cooperatively regulate distinct pathways in PD (Figure 7A). IHC indicated extensive TH loss when each was eradicated, and DA neurons in the SNpc were largely wiped out when both were knocked down (Figure 7C,D). Consistent with these findings, p‐S129 α‐Syn signals were increased when ALDH1A1 or both were depleted (Figure 7E,F). IB analysis validated that Sox6 and ALDH1A1 were significantly reduced by their shRNAs, and the statistical analysis shows that the expression of the Sox6 protein decreased by 46%, and the expression of the ALDH1A1 protein decreased by 57%. As a result, Satb1 and COX IV were abated when either Sox6 or ALDH1A1 was knocked down, and the maximal effect took place when both were exterminated. These were associated with elevated p‐S129 α‐Syn and reduced TH activities (Figure 7G,H). Subsequently, DOPAL levels were significantly increased, and DA concentrations were substantially reduced (Figure 7I). Hence, inactivation of Sox6 and ALDH1A1 by AEP cleavage in DA neurons in the SN triggers DA neuronal loss and motor deficits.

## 3 Discussion

**Figure 8:**
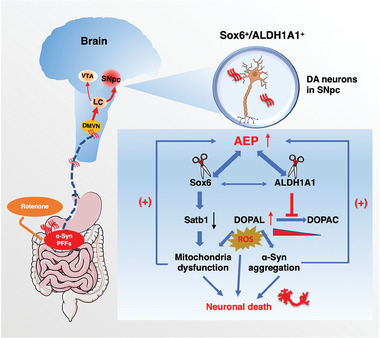
Cleavage of ALDH1A1 and Sox6 by AEP contributes to the vulnerability of dopaminergic neurons in PD. The Gut‐to‐brain propagation of pathologic α‐Syn via vagus nerve‐DMVN‐LC‐SN initiates PD. SNpc DA neurons form stronger connections with the LC than the VTA. AEP is subsequently activated by α‐Syn fibrils in the SNpc, leading to cleavage of Sox6 and ALDH1A1 in SNpc DA neurons. Consequently, Satb1 reduction and DOPAL elevation due to Sox6 and ALDH1A inactivation elicit ROS production and dopaminergic loss, contributing to the vulnerability of dopaminergic neurons in Parkinson's disease. (+) A positive feedback loop.

Defining the molecular factors that make SNpc DA neurons more vulnerable to degeneration in PD is an exciting yet challenging area of research with great potential for new therapies. To date, only a few genes exhibit expression differences between these two neuron groups, and the variations are typically small to moderate. The proteins identified thus far are not exclusively expressed in SNpc or VTA DA neurons but are generally enriched in only one group.^[^ ^28^ ^]^ Sox6 and ALDH1A1 have been identified as target proteins for the susceptibility of dopaminergic neurons in PD,^[^ ^8^, ^29^ ^]^ however, the mechanisms of how Sox6 and ALDH1A1 mediate the onset of PD remain unclear. In the current study, we identified that both Sox6 and ALDH1A are substrates of AEP, which primarily cleaves them at the N446 and N220 residues, respectively, abrogating their protective functions in DA neurons in the ventral tier of the SNpc. Interestingly, α‐Syn fibrils are propagated from the gut to the DMVN and then to the LC in the brain stem, from which the pathological fibrils preferentially spread to the SNpc versus the VTA via neural circuitry. Consequently, α‐Syn fibrils trigger AEP activation, which, in turn, elicits DA neural cell death (**Figure** **8**). These observations are in line with our previous reports that α‐Syn fibrils activate AEP, triggering DA neuronal death.^[^ ^21^, ^30^ ^]^

In our previous study, we showed that gut injected α‐Syn N103 fibrils propagate to the brain stem along vagus nerve to the DMVN to LC and then to SNpc.^[^ ^21^ ^]^ In PD, one of the initial areas to undergo neurodegeneration is the LC,^[^ ^31^ ^]^ moreover, we have reported that norepinephrine metabolite DOPEGAL in the LC activates AEP and pathological Tau aggregation in the LC.^[^ ^32^ ^]^

It has been suggested that the defining characteristic(s) responsible for neurodegeneration must be present in all affected neurons and absent in neurons that either do not degenerate or only do so much later in the disease.^[^ ^33^ ^]^ We show that the upstream triggers, including both α‐Syn fibrils and active AEP, predominantly reside in DA neurons in the SNpc but not the VTA (Figures 3, 4, 5). This groundbreaking finding may shed light on the selective vulnerability of DA neurons in these two regions. This study provides strong support for new drug targets and developing potential neuroprotective therapeutic approach via blocking AEP.

A severe decrease in ALDH1A1 and neurodegeneration in ventral ALDH1A1‐positive DA neurons is found in PD postmortem tissues. Interestingly, ALDH1A1 expression is also suppressed in α‐SNCA Tg mice. Deletion of ALDH1A1 aggravates α‐Syn‐triggered DA neuronal loss and α‐Syn aggregation, whereas both ALDH1A1‐null and control DA neurons are susceptible to MPTP and other triggers.^[^ ^5a^ ^]^ These observations are in alignment with our findings that inactivation of ALDH1A1 results in elevated DOPAL, which elicits massive DA neuronal death (Figure 7I). Moreover, we have reported that DOPAL elevation activates AEP and triggers DA neuronal death in PD.^[^ ^18^, ^34^ ^]^ Interestingly, DOPAL triggers α‐Syn aggregation and neurotoxicity via aldehyde covalent adducts.^[^ ^35^ ^]^ In contrast, ALDH1A1 overexpression preferentially protects against α‐Syn–mediated DA neurodegeneration but does not alleviate cortical neuronal death.^[^ ^5a^ ^]^ Again, these results are consistent with our findings with AEP‐resistant Sox6 N336/446A and ALDH1A1 N220A mutants (Figure 6). ALDH1A1 mediates a non‐canonical GABA synthesis pathway from γ‐aminobutyraldehyde in midbrain dopaminergic neurons to GABA, in addition to DAPAL metabolism.^[^ ^36^ ^]^ Cleavage of ALDH1A1 by AEP may block γ‐aminobutyraldehyde oxidation to carboxylic acid, blunting the GABA synthesis. This γ‐aminobutyraldehyde accumulation may also attribute to DA neurotoxicity. Conceivably, these two highly oxidative and unstable compounds (DOPAL and γ‐aminobutyraldehyde) may jointly facilitate DA neuronal cell death.

Using single‐cell gene expression profiling, Poulin et al. identified multiple molecularly distinct dopamine neuron subtypes and localized them in the adult mouse brain.^[^ ^4^ ^]^ The ALDH1A1‐positive subtype in the substantia nigra is especially vulnerable in the MPTP model of PD. Using translatome‐regulatory network analysis, Greengard et al. identified neurodegenerative factors in PD and disclosed that DNA‐binding protein Satb1 and palmitoyltransferase Zdhhc2 are more highly expressed in SNpc DA neurons than in VTA DA neurons.^[^ ^13^ ^]^ Knockdown of Satb1 mimics the effect of MPTP, triggering a reduction of DA neurons in the SNpc. Hence, the enrichment of Satb1 in adult SNpc DA neurons reflects an intrinsic defense mechanism that prevents neurodegeneration.^[^ ^13^ ^]^ Our study reveals that Sox6 regulates Satb1 expression (Figure S6I–J, Supporting Information), and cleavage of Sox6 by AEP strongly attenuates Satb1 levels in SH‐SY5Y cells, triggering mitochondrial dysfunction (Figures 4, 5, Figure S7G,H, Supporting Information), which is in agreement with a previous report that Satb1 is implicated in protecting the mitochondria.^[^ ^14^ ^]^

One of the most recognized examples of heterogeneity among SNpc DA neurons is the greater vulnerability of ventral SNpc DA neurons compared to dorsal ones. Unraveling the molecular mechanisms behind this differential vulnerability in PD has been particularly challenging.^[^ ^28^ ^]^ Sox6 and ALDH1A1 expression defines a population of neurons that are more ventrally located.^[^ ^5^, ^8^, ^20^ ^]^ The discovery that AEP protease selectively cleaves both Sox6 and ALDH1A1, which suppresses neuroprotective Satb1, induces mitochondrial dysfunction, and elevates toxic DOPAL, which subsequently triggers α‐Syn aggregation and DA neurodegeneration in the ventral tier of the SNpc, provides insight into the molecular mechanism responsible for the distinct vulnerability of DA neurons in the SNpc versus the VTA. This critical pathway regulating the differential susceptibility of DA neurons to degeneration offers significant potential for identifying new drug targets and developing effective neuroprotective therapies. Conceivably, AEP inhibitors may act as an innovative disease‐modifying intervention for treating PD.

## 4 Experimental Section

### Mice and Cell Lines

C57BL/6J mice at different ages (two months, three months, eight months, 17 months, and 25 months) were obtained from Aniphe Biolaboratory Inc. SNCA A53T transgenic mice were originally purchased from the Jackson Laboratory (JAX Stock No. 02 3837). Three‐month‐old SNCA A53T transgenic mice were used in the experiment. DAT‐Cre mice (JAX Stock No: 0 06660), ChaT‐Cre mice (JAX Stock No: 0 06410), TH‐Cre mice (JAX Stock No: 0 08601) were used in the current study. AEP‐knockout mice on a mixed 129/Ola and C57BL/6 background were generated as previously reported. All the mice were bred in specific pathogen‐free facilities at the Shenzhen Institute of Advanced Technology (SIAT), Chinese Academy of Sciences (CAS), maintained on a 12‐hour light/dark cycle with unrestricted access to water and food. All animal experiments followed guidelines of the Institutional Animal Care and Use Committee of Shenzhen Institutes of Advanced Technology, Chinese Academy of Sciences (LLSQ2202140006). HEK293 cells, HEK293 FT cells, and α‐Syn–HEK293 cells were cultured in DMEM supplemented with 10% FBS at 37 °C in an atmosphere containing 5% CO_2_. SH‐SY5Y cells (ATCC, CRL‐2266) were cultured in advanced DMEM/F12 (Gibco) supplemented with 10% FBS(Gibco), penicillin‐streptomycin (Gibco, 2 321 153).

### Plasmids

pcDNA3.1‐His‐Sox6 Full length was purchased from Genscript Biotech Corp, China. GST‐ALDH1A1, GST‐ALDH1A1(1‐220, 221–501) fragments, pcDNA3.1‐His Sox6 (1‐336, 337–808, 1–446, 447–808) fragments were generated using the In‐Fusion HD cloning Kit (Yeasen). His‐Sox6 N336A, His‐Sox6 N446A, His‐Sox6 N336A/N446A, GST‐ALDH1A1 N220A, GST‐ALDH1A1 N389A, GST‐ALDH1A1 N220A/389A were generated using the Fast Site‐Directed Mutagenesis Kit (KM101, TIANGEN BIOTECH (BEIJING) CO., LTD.). Myc‐AEP, Myc‐AEP C189S (Ye lab). SiRNA control and SiSox6 plasmids were purchased from GenePharma. Sox6‐Homo‐633: sense (5′‐3′): GGAAAUGACUCGGACUGAATT; antisense (5′‐3′): UUCAGUCCGAGUCAUUUCCTT. Sox6‐Homo‐780: sense (5′‐3′): GCAGCUCUCCACCAUGAUUTT; antisense (5′‐3′): AAUCAUGGUGGAGAGCUGCTT. Sox6‐Homo‐1162: sense (5′‐3′): GCUUCUGGACUCAGCCCUUTT; antisense (5′‐3′): AAGGGCUGAGUCCAGAAGCTT.

### Viral Vectors

For the animal experiment, rAAV2/9‐TH‐GFP‐WPRE‐pA, AAV2/9‐TH‐His‐Sox6 N336A/N446A‐2a‐EGFP‐WPRE‐PA, AAV2/9‐TH‐6HA‐ALDH1A1 N220A‐2a‐EGFP‐WPRE‐PA, AAV2/9‐TH‐His‐Sox6 447‐808‐2a‐EGFP‐WPRE‐PA, AAV2/9‐TH‐GST‐ALDH1A1 221‐501‐2a‐mcherry‐WPRE‐PA, AAV2/9‐U6‐shALDH1A1‐CMV‐EGFP‐T2A‐Puro‐WPRE (5′‐GCACCATGGATGCTTCAGAGA‐3′), AAV2/9‐U6‐shSox6‐CMV‐EGFP‐T2A‐Puro‐WPRE (5′‐GCAAAGAATGGAGTCAGAGAA‐3′), AAV2/9‐U6‐shRNA(scramble)‐CMV‐EGFP‐WPRE virus were purchased from the BrainVTA company.

### Reagents

AEP substrate Z‐Ala‐Ala‐Asn‐AMC (Bachem, 4 033 201). The recombinant AEP was purchased from SinoBiological Company, Beijing, China. Lipofectamine 3000 (Invitrogen, L3000008). DA (Sigma‐Aldrich, PHR1090‐1G). DOPAL (Cayman, 18448–50mg). LDH‐Glo™ Cytotoxicity Assay (Promega, J2380). ALDH Activity Assay Kit (Colorimetric, ab155893‐100 tests). HisPur™ Ni‐NTA Purification Kit (Therm, 88 229). Mouse and Rabbit Specific HRP/DAB (ABC) Detection IHC kit (ab64264). Mouse and Rabbit Specific HRP/DAB IHC Detection Kit (ab236466). Fast Site‐Directed Mutagenesis Kit (TIANGEN, KM101). Hieff Clone® Universal One Step Cloning Kit (10922ES50, YEASEN). MitoTracker® Red CMXRos (Invitrogen, M7512). Reactive Oxygen Species Assay Kit (50101ES01, YEASEN). Neurobasal Medium (Gibco, 21 103 049). Rotenone (Sigma‐Aldrich, R8875‐1G). NE‐PER Nuclear and Cytoplasmic Extraction Reagents kit (Thermo, 78 835).

### Human Tissue Samples

Postmortem brain samples were dissected from five control cases and six PD cases obtained from the Emory Alzheimer's Disease Research Center (No.1RF1AG051538‐01A1). Informed consents were obtained from all subjects. The study was approved by the Committee of Shenzhen Institutes of Advanced Technology, Chinese Academy of Sciences (LLSQ2202140006). PD and PDD cases were clinically diagnosed and neuropathologically confirmed. The details of human samples are shown in Table S2 (Supporting Information).

### Tracing Virus and Stereotaxic Surgeries

The information on tracing virus used in the current study is below: for the TH‐Cre mice: AAV‐EF1α‐DIO‐EGFP‐T2A, AAV‐EF1α‐DIO‐RVG, RV‐EnvA‐ΔG‐mCherry; for DAT‐Cre mice: rAAV‐EF1α‐DIO‐EGFP‐T2A‐TK(HSV), H129△TK‐CAG‐LSL‐tdTomato; for the Chat‐Cre mice: AAV2/9‐hSyn‐DIO‐tdTomato‐T2A‐synaptophysin‐EGFP‐WPRE‐pA. The titer of the viruses ranges from 1 to 5 × 10^13^ GC mL^−1^. Rodents underwent anesthesia with 2‐4% isoflurane and were placed in a stereotaxic apparatus (RWD, 68 019). Standard protocol was performed as previously described. Injections were performed using the NANOJECT III system, with a virus volume of 200 nL administered to the VTA, SNpc, LC, or DMVN. The following coordinates were used for virus injection: VTA (AP −3.2 mm; ML −0.3 mm; DV −4.4 mm);SNpc (AP −3.2 mm; ML −1.4 mm; DV −4.15 mm), DMVN (AP: −7.48 mm; ML:0.25 mm; DV: 4.5 mm).

### Cell Culture and Transfection

Cells were transfected with plasmids encoding His‐Sox6 full length and mutants, GST‐ALDH1A1 full length, and mutants with Myc‐AEP or Myc–AEP C189S using the Lipofectamine 3000. Cultivation of the SH‐SY5Y, HEK293, and α‐Syn‐HEK293 cell lines was conducted in DMEM supplemented with 10% FBS at 37 °C in an atmosphere containing 5% CO_2_. Primary cortical neuron isolation and culture followed established protocols from prior studies. These neurons underwent various interventions in vitro (DIV) 10 and were then utilized for immunofluorescence analyses by the 14^th^ day. Compound CP#11A was prepared in a DMSO solution for storage. In every group, the ultimate dilution of either DMSO or pure ethanol present in the medium did not exceed 0.1%.

### Nuclear and Cytoplasmic Extraction

Cells underwent lysis and subsequent treatment using a commercial nuclear protein isolation kit as per the guidelines provided by the supplier (Thermo#78 835). To evaluate the presence of histone H3, indicative of nuclear content, western blot analysis was performed.

### Primary Neuron Cultures

Cortical neurons were cultured from Sprague‐Dawley rat embryos on day 14 by excising the cortex and dissociating it with 0.05% trypsin for 10 min at 37 °C. The reaction was halted with 20% fetal bovine serum (FBS), and the mixture was centrifuged at 1000 × g for 5 min. Neurons were plated on coverslips in 6‐well plates coated with 100 µg mL^−1^ poly‐D‐lysine, using a medium of DMEM/F12 with 10% FBS. After 6 h, the medium was replaced with Neurobasal medium (Gibco) supplemented with B‐27 and GlutaMAX, and cultured at 37 °C with 5% CO_2_. To assess the effects of rotenone on primary dopaminergic neurons, 200 nM rotenone was added to cultures at 7 days in vitro (DIV 7) for 24 h. After treatment, neurons were fixed in 4% formaldehyde, permeabilized, and immunostained with primary antibodies against TH, Sox6, and ALDH1A1.

### LDH Assay

LDH activity was measured following the Promega LDH assay kit protocol (J2380). First, thaw the LDH detection enzyme mix and reductase substrate at room temperature. Prepare the LDH Detection Reagent by combining 50 µL of the enzyme mix with 0.25 µl of the substrate and inverting gently five times. Make the LDH storage buffer from stock solutions to achieve 200 mM Tris‐HCl (pH 7.3), 10% glycerol, and 1% BSA. Dilute the samples in this buffer, then transfer 50 µL of the diluted sample into a 96‐well opaque assay plate. Add 50 µL of the LDH detection reagent and incubate for 60 minutes at room temperature. Finally, record the luminescence.

### ALDH Activity Assay

The ALDH assay was conducted according to kit instructions (ab155893). For the standard curve, 0, 2, 4, 6, 8, and 10 µL of the standard were added in duplicate to a 96‐well plate, achieving final concentrations of 0, 2, 4, 6, 8, and 10 nmol per well, with each well adjusted to 50 µL using Assay Buffer XXVIII. Cells (1 × 10^6^) were homogenized in 200 µL of ice‐cold Assay Buffer for 10 min, then centrifuged at 12 000 rpm for 5 min. 50 microliters of the supernatant were transferred to a 96‐well plate and brought to 50 µL with Assay Buffer. After mixing, 50 µL of Reaction Mix was added, and the plate was incubated for 5 min at room temperature. Optical density (OD) was measured at 450 nm initially (A1 & A1B) and again after 20–60 min (A2 & A2B), based on ALDH activity.

### Preparation of α‐Syn PFFs

Recombinant mouse α‐Syn PFFs were prepared as described previously.^[^ ^37^ ^]^ In brief, α‐Syn monomers were incubated in 1.5 mL microcentrifuge tubes in PBS (2 mg mL^−1^) at 37 °C for 7 days with shaking at 1000 rpm. The formation of amyloid structures was confirmed using Thioflavin T fluorescence. The α‐Syn PFFs were then stored at –80 °C and sonicated at 50 Hz in an ice‐water bath for 30 min prior to transduction.

### Intestinal Intramuscular Injection of α‐Syn PFFs

Three‐month‐old mice were anesthetized using isoflurane (2–4%), Figure S1D–E (Supporting Information) provides a schematic of how the gastrointestinal α‐Syn PFFs injection experiment was performed. Detailed experimental procedures were performed as previously described.^[^ ^38^ ^]^ The stomach was injected at two sites with a total of 5 µg of ATTO550‐labeled α‐Syn PFFs (2 mg mL^−1^, 2.5 µL per site), and the duodenum was similarly injected with another 5 µg, totaling 10 µg of α‐Syn PFFs in each region. Control mice received the same volume of ATTO550 alone at the same sites. After injections, the animals were sutured and returned to standard housing. Mice were sacrificed at one week, one month, and three months post‐injection.

### In Vitro Sox6 or ALDH1A1 Cleavage Assay

To assess the in vitro cleavage of Sox6 or ALDH1A1 by AEP, HEK293T cells were transfected with His‐Sox6 or GST‐ALDH1A1 plasmids using PEI. 36 h post‐transfection, cells were collected, washed with PBS, lysed in the buffer (50 mM sodium citrate, 5 mM dithiothreitol (DTT), 0.1% CHAPS, and 0.5% Triton X‐100, pH 7.4), and centrifuged at 14 000g for 10 min at 4 °C. The supernatant was incubated with rAEP at either pH 7.4 or pH 6.0 at 37 °C for 5–15 min. For purified Sox6 or ALDH1A1 fragment cleavage by AEP, His‐tagged Sox6 or GST‐ALDH1A1 was purified using Ni‐NTA or glutathione beads. The purified proteins were incubated with rAEP in AEP buffer (50 mM sodium citrate, 5 mM DTT, 0.1% CHAPS, and 0.5% Triton X‐100, pH 6.0) for 10 min. The samples were boiled in 5 x SDS loading buffer and analyzed via immunoblotting.

### Antibodies

Generation of antibodies that specifically recognize the AEP‐generated Sox6 or ALDH1A1 fragments. The anti‐Sox6 N336, anti‐Sox6 C447 and ALDH1A1 N220 antibodies were generated by immunizing rabbits with the peptide QKGHVSHPQIN (Sox6 N336), LFPASKTSPVNLPNKS (Sox6 C447), TVVVKPAEQTPLTALHVASLIKEAGFPPGVVN (ALDH1A1 N220). The antiserum was pooled, and its titer against the immunizing peptide was determined using ELISA. The highest dilution that produced a positive response with the chromogenic substrate for horseradish peroxidase was 1:50000. Validation of the antiserum's specificity was extended by conducting both Western blot and immunohistochemical analyses.

### Mass Spectrometry Analysis

Protein samples were digested in‐gel with trypsin for mass spectrometry analysis. Peptides were resuspended in a loading buffer (0.1% formic acid, 0.03% trifluoroacetic acid, and 1% acetonitrile) and loaded onto a 20‐cm nano‐HPLC column packed with Reprosil‐Pur 120 C18‐AQ beads. Elution was performed over a 2‐hour reverse‐phase gradient (4–80% buffer B) using a NanoAcquity UPLC system. Peptides were ionized at 2.0 kV using a nano‐ESI source on a hybrid LTQ XL Orbitrap mass spectrometer. MS spectra were acquired at 120000 resolution, and tandem mass spectrometry (MS/MS) spectra were obtained via electron‐transfer dissociation with supplemental activation. Proteome Discoverer 2.0 was used to search MS/MS spectra for AEP‐cleavage sites in human Sox6 or ALDH1A1 against the complete human proteome database, allowing cleavage at glutamates and asparagines. Peptide spectral matches were filtered to a false discovery rate of <1%, and all MS/MS spectra of putative cleavage sites were manually inspected.

### AAV Infection in SNpc

rAAV‐GFP, AAV‐Sox6 FL, AAV‐His‐Sox6 N336A/N446A, AAV‐ALDH1A1 FL, AAV‐ALDH1A1 N220A, AAV‐Sox6 447–808, AAV‐ALDH1A1 221–501, AAV‐shALDH1A1, AAV‐shSox6, AAV‐shRNA(scramble) virus under the control of TH promoter was injected into blateral SNpc of SNCA A53T mice through Stereotaxic surgeries. The following coordinates were used for virus injection: SNpc (AP ‐3.2 mm; ML ±1.4 mm; DV ‐4.15 mm).

### Behavioral Tests: Rotarod Test

Mice were trained on a spinning rod for 3 consecutive days, with 3 sessions per day lasting 5 min each. The rod accelerated from 5 to 25 rpm, increasing by 1 rpm every 5 s, with a 30‐minute rest between sessions. Testing occurred 1 h after the final training session, recording the time until the mice fell, up to a maximum of 300 s per trial. Pole Test: Mice were placed head‐up on a vertical wooden pole (45 cm long, 1 cm diameter) and allowed to descend freely. They underwent training for 2 days, 3 times daily. Testing began after the last training session, measuring the time from turning to descent, with a maximum cutoff of 30 s per trial. Wire Hang Test: Mice were placed on a 0.5 cm wire mesh, which was inverted and suspended 1 meter above a cage for 2 min. Mice that lost grip fell into a soft bedding‐filled cage, and the time to fall was recorded for each mouse.

### AEP Activity Assay

Tissue homogenates or cell lysates (10 µg) were incubated in 200 µL of assay buffer (20 mM citric acid, 60 mM Na₂HPO₄, 1 mM EDTA, 0.1% CHAPS, and 1 mM DTT, pH 6.0) with 20 µM AEP substrate Z‐Ala‐Ala‐Asn‐AMC (Bachem). The released AMC from substrate cleavage was quantified by measuring fluorescence at 460 nm using a plate reader at 37 °C for 1 h in kinetic mode.

### Detection of Intracellular ROS Generation

Reactive oxygen species (ROS) generation in SH‐SY5Y cells was measured using 2,7‐dichlorodihydrofluorescein diacetate (DCFH‐DA). Approximately 5 × 10⁵ SH‐SY5Y cells per well were cultured in 6‐well plates for 24 h, followed by various treatments. After 30 min of ultrasound irradiation, 10 µM DCFH‐DA was added and incubated for 30 min at 37 °C in the dark. The wells were washed three times with PBS, and fluorescence intensity was observed using a fluorescence microscope.

### Mitochondrial Membrane Potential Experiment

MitoTracker Red CMXRos, a mitochondrial red fluorescent probe, was used to label biologically active mitochondria and detect mitochondrial membrane potential (MMP). Cells were seeded in a 12‐well plate. After treatment, the cells were incubated with MitoTracker Red CMXRos staining solution (0.2 µM) at 37 °C for 30 min. The staining solution was then replaced with fresh cell culture medium, and the cells were observed using a fluorescence microscope. The MMP was calculated based on the fluorescence intensity ratio.

### LC‐MS Analysis to Detect Dopamine Metabolites

The extraction solution consisted of 1% acetic acid and 100 µg mL^−1^ Vitamin C in methanol. For human substantia nigra (SN) and mouse striatum and SN tissues, 10 mg of each sample was placed in a 1.5 mL tube. After adding 200 µL of the extraction solution, samples were homogenized at 40 Hz for 30 s at 4 °C, vortexed for 15 min, and centrifuged at 12 000 rpm for 15 min at 4 °C. A 100 µL aliquot of the supernatant was then transferred to an autosampler vial for liquid chromatography‐tandem mass spectrometry (LC‐MS/MS) analysis, with a 1 µL injection volume. UHPLC separation was conducted using an Agilent 1290 Infinity II series system equipped with an Agilent ZORBAX Eclipse Plus C18 column. Dopamine and DOPAL concentrations in mouse brain were reported in nanograms per milligram of tissue, while human serum samples were reported in micromolar per liter.

### Western Blot Analysis

Cells and brain tissue samples were washed with 1x PBS and lysed in the buffer (50 mM Tris, pH 7.4, 40 mM NaCl, 1 mM EDTA, 0.5% Triton X‐100, 1.5 mM Na3VO4, 50 mM NaF, 10 mM sodium pyrophosphate, 10 mM sodium β‐glycerophosphate, supplemented with protease inhibitors) at 4 °C for 30 min. The lysates were centrifuged at 15 000 rpm for 15 min. The supernatant was boiled in 5 x SDS loading buffer. After SDS‐PAGE, samples were transferred to a PVDF membrane, which was blocked with TBST containing 5% nonfat milk at room temperature for 1h. The membrane was incubated with the primary antibody overnight at 4 °C, followed by secondary antibody incubation at room temperature for 1.5 hours. After washing with TBST, the membrane was developed using the enhanced chemiluminescence (ECL) detection system. Antibodies used for western blotting are listed in Table S1 (Supporting Information).

### Immunofluorescence

For immunostaining, free‐floating 30 µm brain sections were used. The samples were blocked in a solution containing 1% BSA and 0.3% Triton X‐100 for 30 min, followed by overnight incubation with primary antibodies (listed in Table S1, Supporting Information) at 4 °C. After washing with PBST, the sections were incubated with Alexa Fluor 488‐, 555‐, and 647‐conjugated secondary antibodies (Invitrogen) for detection. DAPI (1 µg mL^−1^) (Sigma) was used to stain nuclei. Coverslips were then mounted on glass slides, and imaging was performed using a confocal microscope (LSM 980, Zeiss). To assess the levels of His, pS129‐α‐Syn, or AEP in TH‐positive neurons, the pS129‐α‐Syn and TH‐positive signals were quantified using ImageJ software.

### Immunochemistry

Mice were anesthetized, sacrificed, and perfused with saline and 4% paraformaldehyde. The brains were isolated, fixed in 4% paraformaldehyde overnight, embedded, and sliced into 4 µm sections. Sections were dewaxed in xylene, rehydrated through an ethanol gradient, and subjected to antigen retrieval in 0.1 M sodium citrate buffer (pH 6.0) at 95 °C for 20 min. For immunohistochemical (IHC) staining, sections were treated with 3% H_2_O_2_ for 10 minutes, blocked with 5% BSA for 30 min, and incubated with primary antibodies overnight at 4 °C. Signals were developed using the Mouse and Rabbit Specific HRP/DAB Detection IHC kit (Abcam, ab64264). The spread of α‐Syn pathology was assessed by analyzing the percentage of neurons with pS129‐positive aggregates. Dopaminergic neurons were counted from substantia nigra sections, with one section taken from every six for TH staining, totaling 20–25 sections for the count.

### Optical Densitometry Analysis

The intensity of TH‐positive fibers in the striatum was quantified using optical densitometry on TH‐stained mouse brain sections from the same striatal area. Sections from the injection side were analyzed, and measurements were conducted using ImageJ software (NIH).

### Quantification and Statistical Analysis

Results were analyzed using GraphPad Prism 8 software. Statistical comparisons were made using Student's t‐test for two groups and one‐way ANOVA with Tukey's multiple‐comparisons test for more than two groups. Data are presented as mean ± SEM, with p values less than 0.05 considered significant.

## Conflict of Interest

The authors declare no conflict of interest.

## Author Contributions

S.N. and B.L. contributed equally to this work. K.Y. and X.Y. conceived the project, designed the experiments, analyzed the data, and wrote the manuscript. S.N. and B.L. designed and performed most of the experiments, analyzed the data, and wrote the manuscript. Z.Q. and Z.X. performed the cell culture and plasmid construction. J.H. and M.W. performed the behavioral experiments. J.R., Z.L., Z.C., X.X., and Y.Z. conducted tracing virus injection and brain tissues preparation. Z.Z., Z.Z., and Z.C. assisted with data analysis and interpretation. All the authors approved the manuscript.

## Supporting information



Supporting Information

## Data Availability

Research data are not shared.
