# Supplementary material for: Sox6 and ALDH1A1 Truncation by Asparagine Endopeptidase Defines Selective Neuronal Vulnerability in Parkinson's Disease
Source: Adv Sci (Weinh). 2024 Nov 21;12(2):2409477. doi: 10.1002/advs.202409477 (PMC11727119; doi:10.1002/advs.202409477)
Supplement: Supplementary file 1 — Supporting Information [file ADVS-12-2409477-s001.docx]

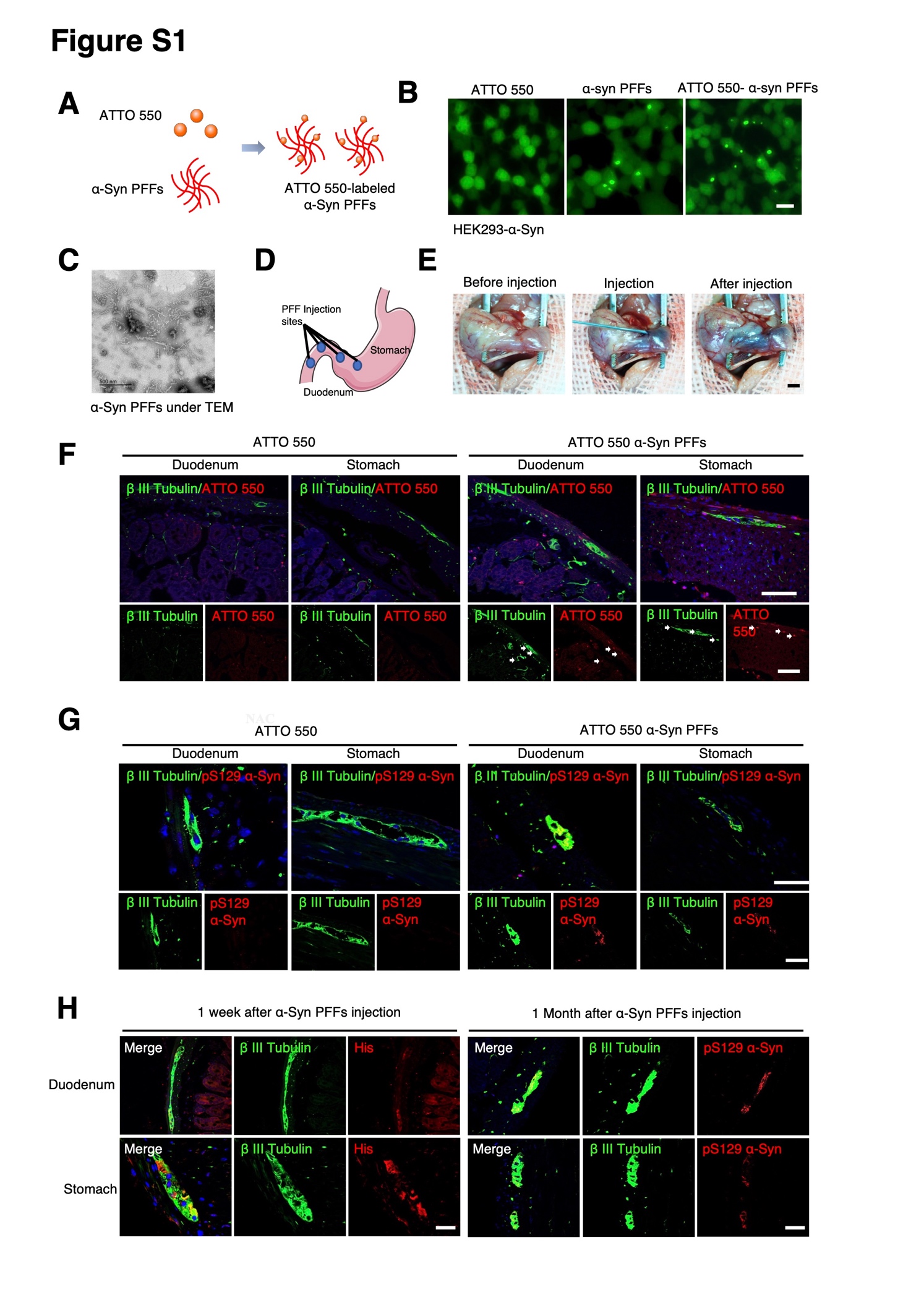


**Figure S1. Spread of pathologic α-Syn from the gut to the brain.**

A) Schematic showing the conjugation of ATTO 550 and His-α-Syn PFFs. B) ATTO550-labeled α-Syn PFFs’ aggregation ability were validated in HEK293-α-Syn stable cells. C) Representative transmission electron microscopy (TEM) images for α-Syn PFFs and ATTO550-conjugated α-Syn PFFs. D, E) Schematic and confirmation of injection sites by trypan blue in the upper duodenum (UD) and pyloric stomach (PS). F) Representative double-immunostaining for ATTO 550 (red) and β III Tubulin (green) in the upper duodenum (UD) and pyloric stomach (PS) after 1-week post-injection. G, H) Representative double-immunostaining for pSer129-α-Syn PFFs (red) and and β III Tubulin (green) in the upper duodenum (UD) and pyloric stomach (PS) after 1-month post-injection.


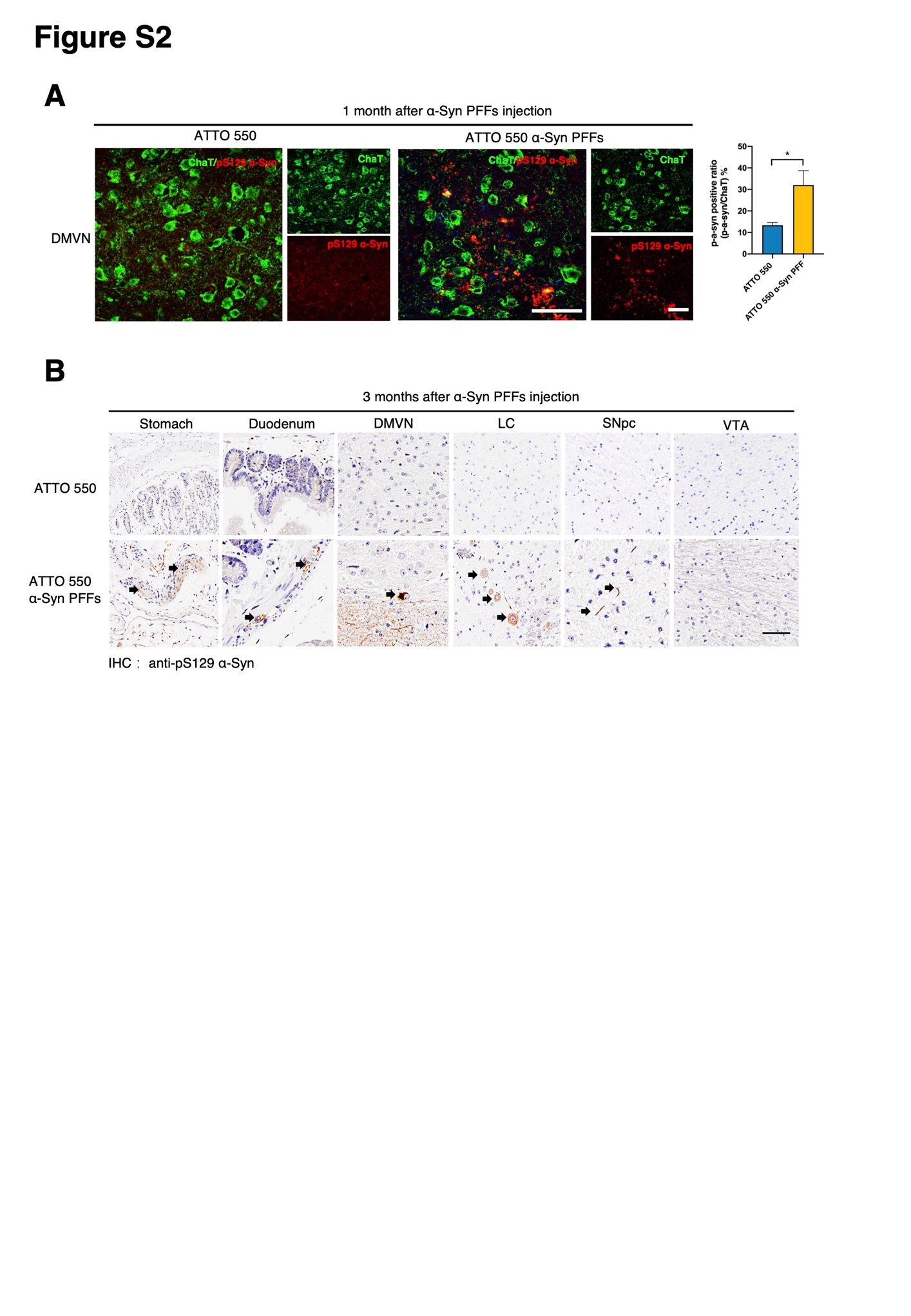


**Figure S2. α-Syn PFFs preferentially spread from the LC to SNpc versus VTA.**

A) Representative image and quantification showed that ATTO550 α-Syn elicited substantial p-S129 α-Syn signals in ChaT neurons in the DMVN. (Scale bars, 20 μm). **P* < 0.05. B) Distribution of pSer129-α-Syn accumulation in stomach, duodenum, DMVN, LC, SNpc and VTA of the mice after three months post-injection. (Scale bars, 100 μm).


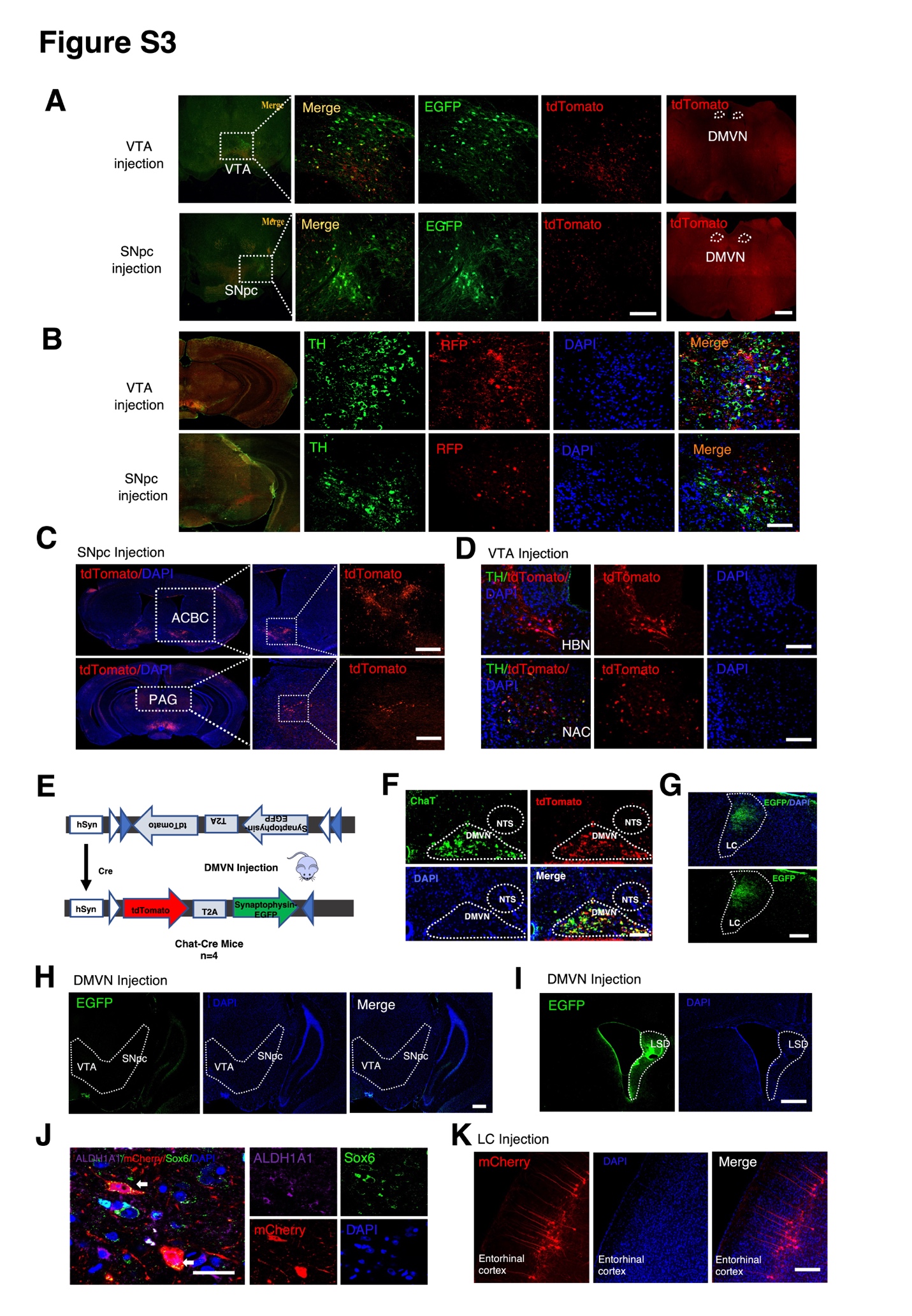


**Figure S3. Neural circuitry mapping study to confirm the connection and projection among DMVN, LC and SNpc/VTA.**

A) Representative double-immunostaining for tdTomato (red) and EGFP (green) in the SNpc and VTA after 1month post-injection. (Left scale bar, 100 μm). There was no detectable projection from either VTA or SNpc to DMVA. (Right scale bar, 400 μm). B) Confirmation of anterograde labeled tdTomato expressed in the DA neurons of VTA and SNpc. (Scale bar, 100 μm). C) SNpc DA neurons projected to other different brains regions including ACBC, PAG (Scale bar, 200 μm). D) VTA DA neurons project to HBN and NAC. (Scale bar, 200 μm). E) Schematic showing the anterograde viral tracing strategy to investigate the direct projection from DMVN to LC with Chat-Cre mice (n=4). F) Representative staining showing the successful expression of tdTomato in ChaT-positive neurons of the DMVN. G) Robust of synaptophysin-EGFP positive projections were observed in the LC area. The result indicates that DMVN Chat-positive neurons make direct connections with LC neurons. H) EGFP-labeled cholinergic neurons (ChaT positive) in the DMVN had no direct connection with DA neurons in the SNpc or VTA area. I) DMVN ChaT neurons projected to other brains regions including LSD. (Scale bar, 200 μm). J) Representative triple -immunostaining for mCherry (red), ALDH1A1 (purple) and Sox6 (green) in the SNpc after 1month post-LC injection. (Scale bar, 20 μm). K) LC neurons also received input from the neurons in the entorhinal cortex. (Scale bar, 100 μm).

**
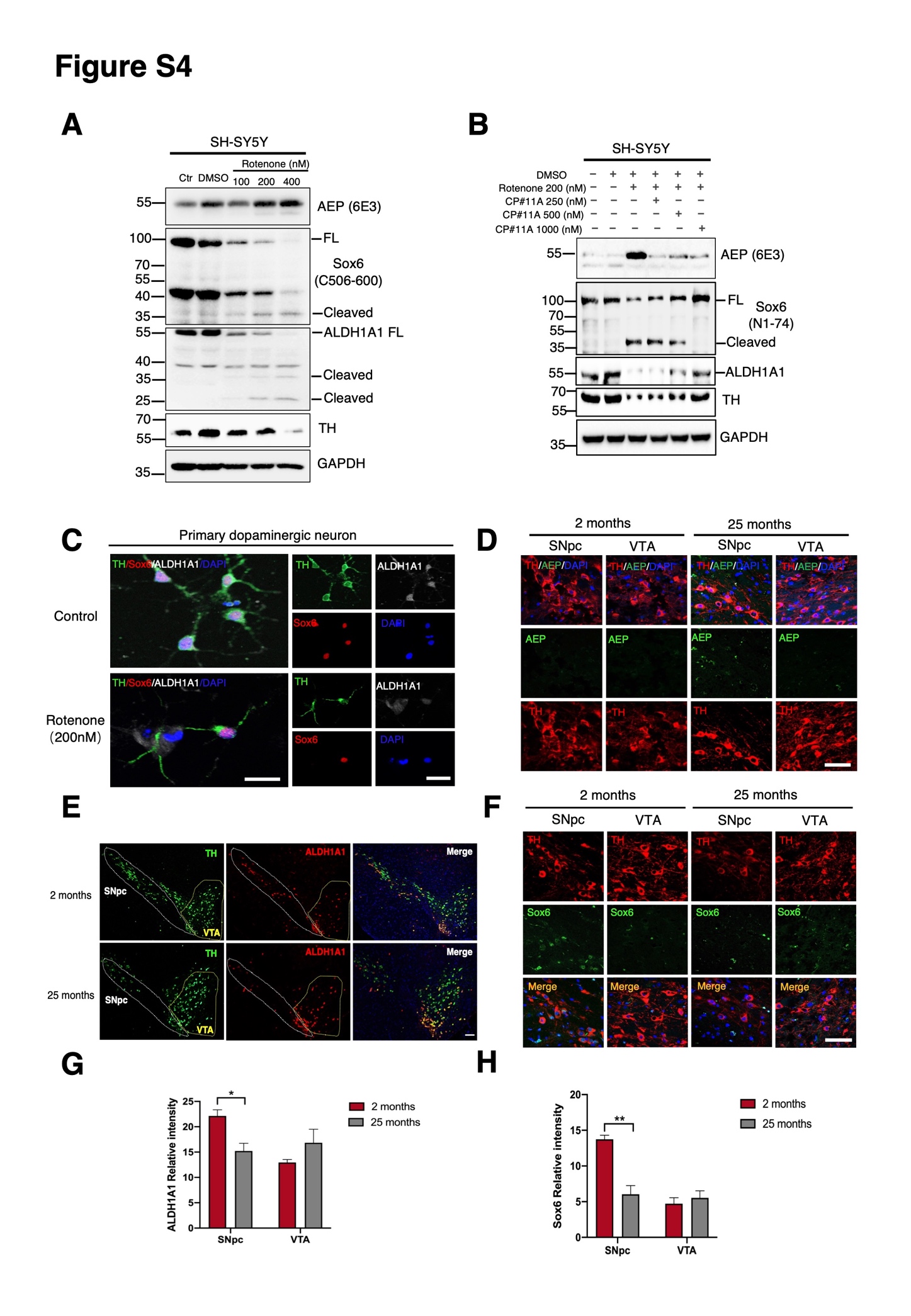
**

**Figure S4. Sox6 and ALDH1A1 were cleaved by active AEP in cells and aged mice.**

A) Representative immunoblots showed AEP cleaved Sox6 and ALDH1A1 in rotenone-treated SH-SY5Y cells. B) Truncation of Sox6 and ALDH1A1 in rotenone-treated SH-SY5Y cells were blocked by AEP inhibitor CP#11A. C) IF analysis showed that both of Sox6 and ALDH1A1 were repressed by rotenone in primary dopaminergic neurons. (Left scale bar, 20 μm). D) Representative staining and quantification of the expressions of AEP in SNpc and VTA DA neurons of the PD model (scale bars, 20 μm). E-H) IF co-staining and quantifications showed that both Sox6 and ALDH1A1 signals were greatly reduced in the SNpc from 25-month-old mice compared to 2-month-old, whereas they remained unchanged in VTA. (Scale bar, 100 μm). **P* < 0.05; ***P* < 0.01.

**
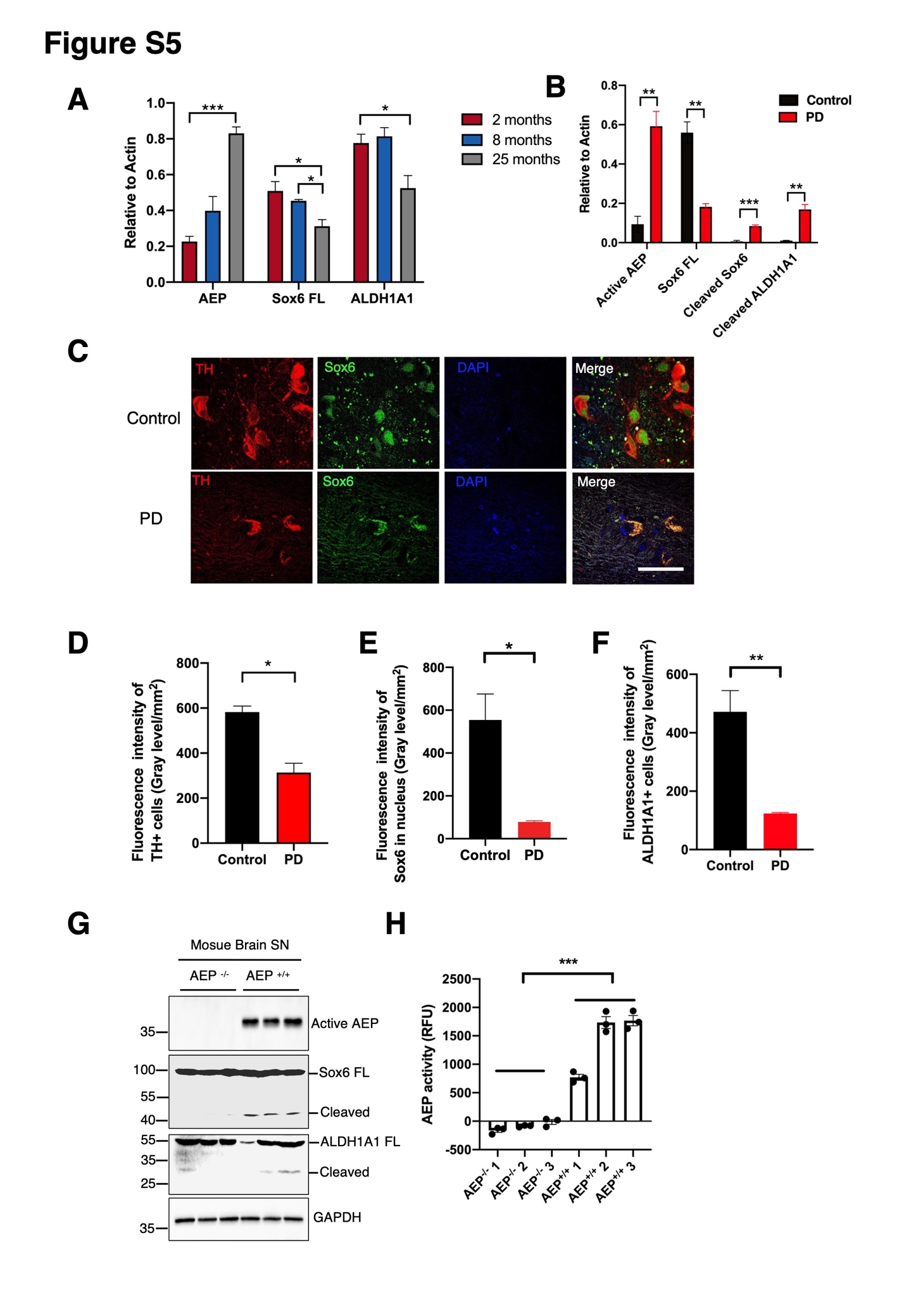
**

**Figure S5. Active AEP in the SNpc cleaves both Sox6 and ALDH1A1.**

A) Quantification of the protein levels of AEP, Sox6 and ALDH1A1 in the SN of aged mice (n=3 per group). B) Quantification of the protein levels of AEP, Sox6 and ALDH1A1 fragments in PD brains compared to healthy controls. (n=3 per group). C) Representative IF staining and quantifications showed the expressions of Sox6 and ALDH1A1 were highly reduced in PD brains, associated with significant TH-positive neuronal loss. (Scale bar, 50 μm). **P* < 0.05; ***P* < 0.01. G, H) Cleavage of Sox6 and ALDH1A1 was completely abated in AEP^-/-^ mice. (n=3 per group). ****P* < 0.001. All data are presented as the mean ± SEM from 3 to 6 independent experiments.

**
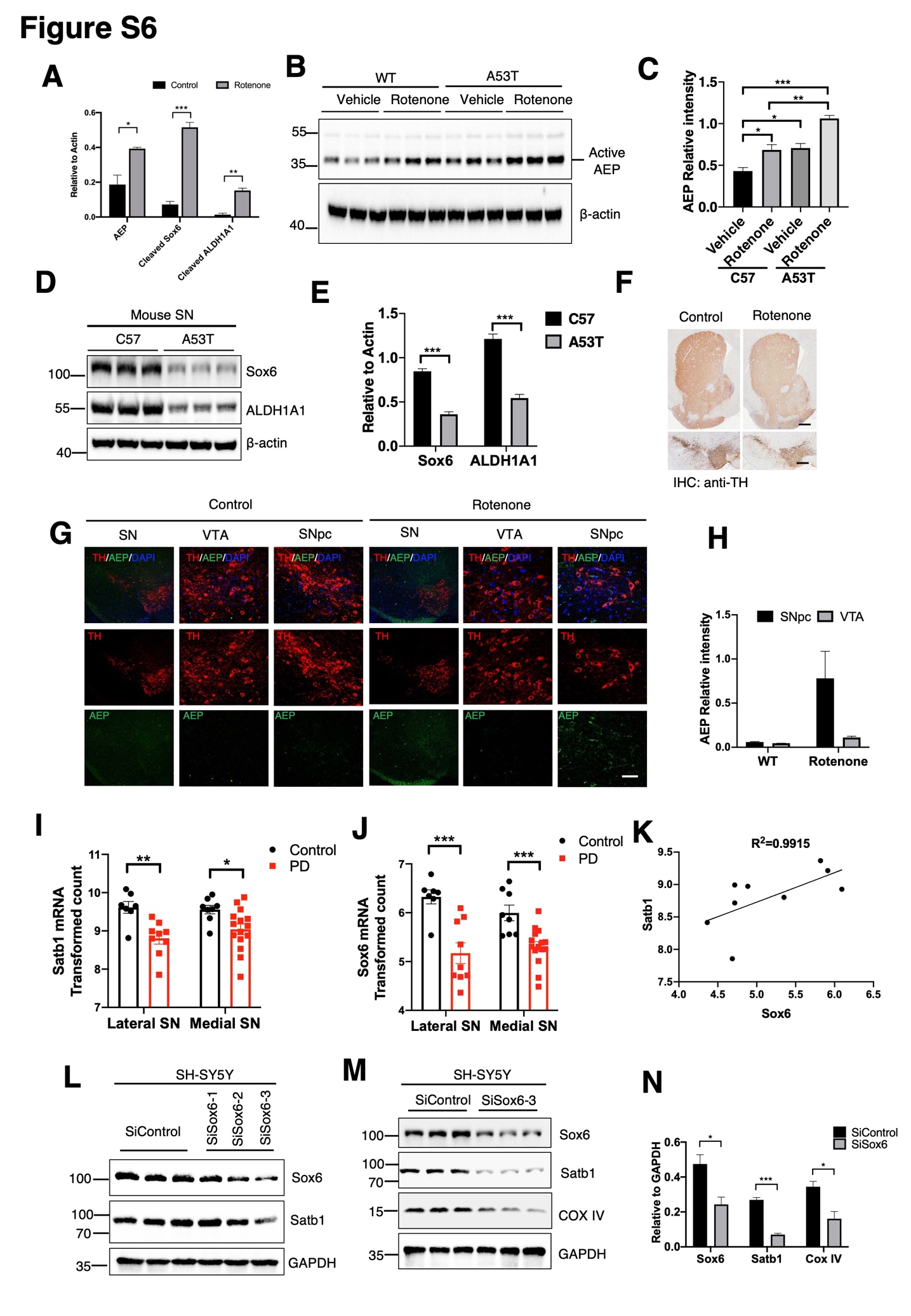
**

**Figure S6. Cleavage of Sox6 and ALDH1A1 in human PD tissues and PD mice, and the correlation between Sox6 and Satb1.**

A) Quantification of the levels of TH, Sox6 and ALDH1A1 in healthy control and PD patient brain sections. B, C) The expression and quantification of active AEP in the SN of rotenone-treated WT and A53T mice (n=3 per group). D, E) The expression and quantification of Sox6 and ALDH1A1 in the SN of WT and A53T mice (n=3 per group). F) Representative TH IHC staining of striatum and SNpc/VTA in rotenone-induced PD mice (n=3 per group). (Scale bars, upper panel 400 μm; lower panel, 100 μm). G) Representative TH/AEP co-staining of striatum and SNpc/VTA in the control and rotenone groups (n=3 per group). (Scale bars, 200 μm). H) Quantification of the intensity of AEP in the mouse SNpc/VTA between WT and Rotenone groups. I-K) GEO DataSet re-analysis showed that Satb1 mRNA were significantly reduced in both lateral and medial SN regions in PD, fitting with decreased Sox6 mRNA levels. L-N) Knockdown of Sox6 in SH-SY5Y cells evidently repressed both Satb1 and COX IV expressions. All data are presented as the mean ± SEM from 3 to 6 independent experiments. **P* < 0.05; ***P* < 0.01; ****P* < 0.001.


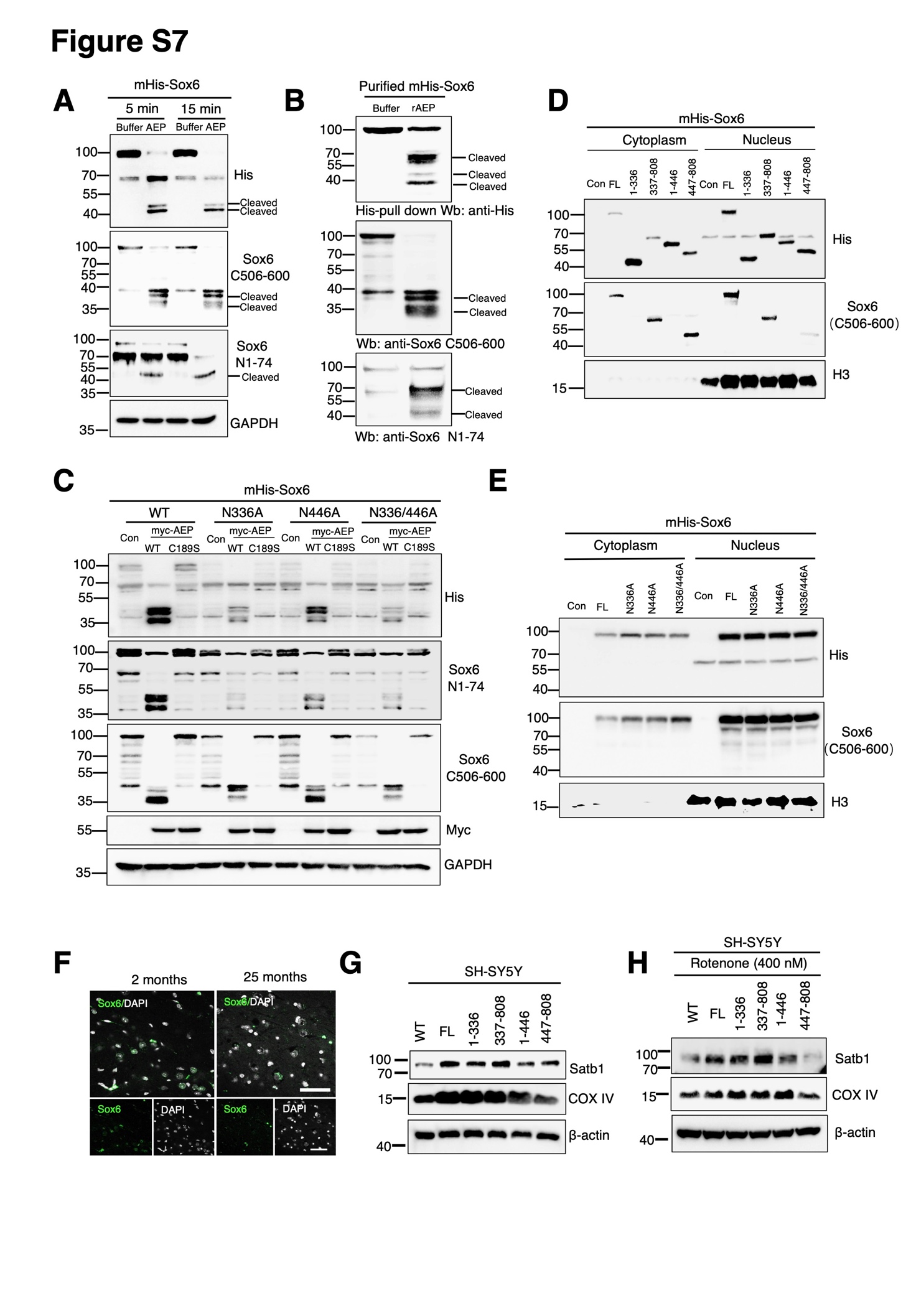


**Figure S7.** **AEP cleaves Sox6 at N336/446 and severely impairs nuclear localization.**

A) HEK293 cells lysates overexpressing His-Sox6 were incubated with rAEP for 5 min and 15 min. Western blot showing the cleavage of Sox6 by recombinant AEP in a time-dependent manner. B) Cleavage of purified His-Sox6 analyzed by immunoblotting. C) N336A or N446A mutant blunt correspondent Sox6 fragmentation, and N336/446A substantially abrogated Sox6 cleavage. D, E) Subcellular fractionation revealed that full-length Sox6 (FL) mainly resided in the nucleus, and N336 or N446 cleavage augmented their cytoplasmic distribution. F) Representative staining showed that Sox6 primarily resided in the nucleus of DA neurons in 2-month-old mice, while some were re-distributed in the cytoplasm in 25-month-old mice. (Scale bars, 100 μm). G, H) Quantification of the levels of Satb1 and COX IV in SH-SY5Y cells overexpressing Sox6 FL and fragments.


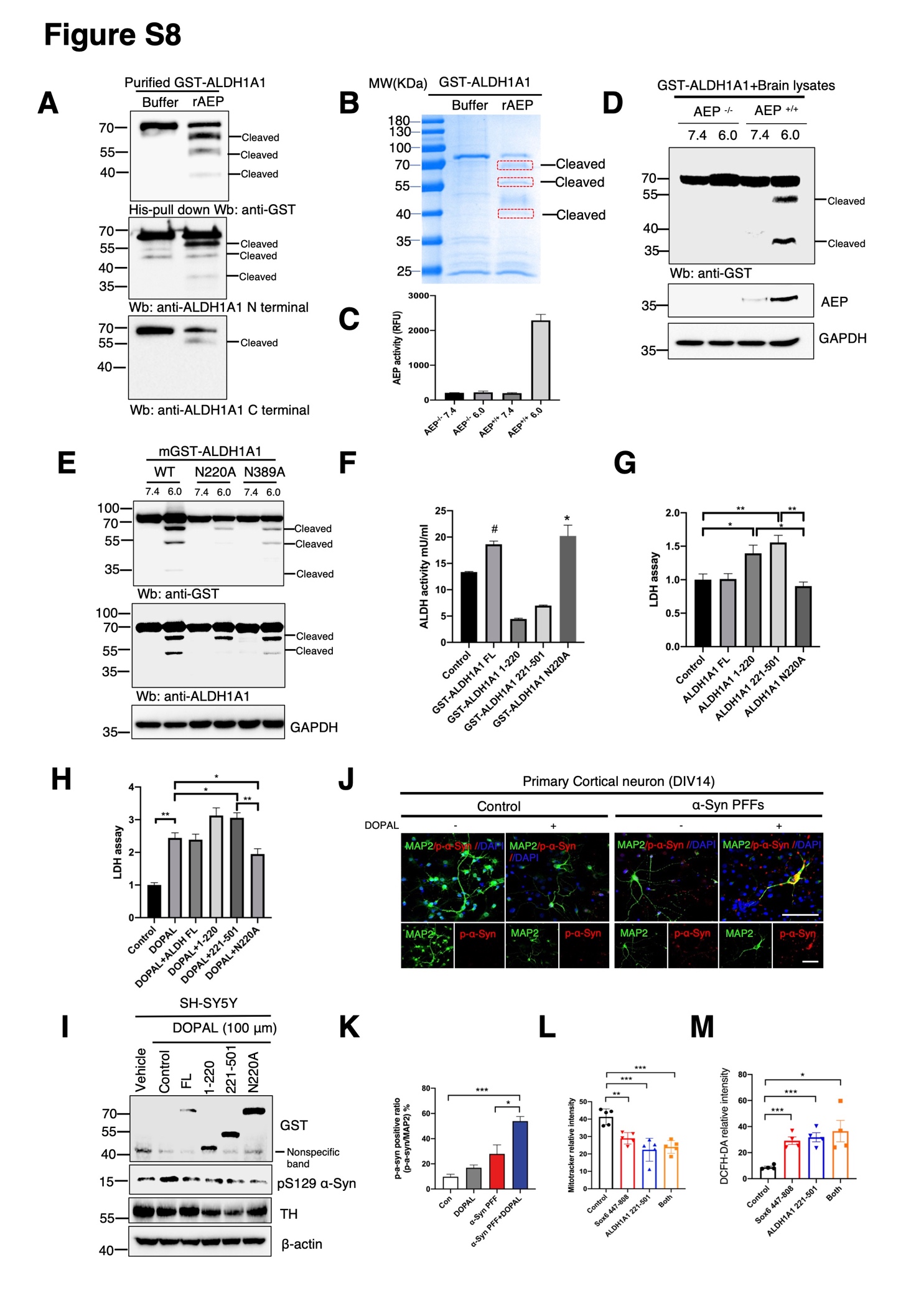


**Figure S8. AEP cleaves ALDH1A1 at N220/389 and impairs its enzymatic activity.**

A) In vitro cleavage assays with GST-ALDH1A1 showed that AEP strongly cleaved it into several fragments. B) Cleavage of purified GST-ALDH1A1 analyzed by Coomassie blue staining. C, D) Knockout of AEP from the brain abolished its fragmentation of ALDH1A1. E) Mutation assay supported that N220 was the predominant cutting site on ALDH1A1. F) ALDH enzymatic assay showed that cleavage of ALDH1A1 by AEP at N220 disrupted ALDH1A1 activity. G, H) LDH assay and representative immunoblots showed that fragmentation of ALDH1A1 enhanced SH-SY5Y cell death, which was further augmented by DOPAL. J, K) DOPAL accelerated α-synuclein phosphorylation and aggregation in primary neuronal cultures. (Scale bars, 20 μm). L, M) Quantification of the levels of ROS and impaired mitochondrial membrane potential in SH-SY5Y cells overexpressing Sox6 447-808 or ALDH1A1 221-501 or their combination. **P* < 0.05; ***P* < 0.01; ****P* < 0.001.

**Table S1. Antibodies**

| Antibody | Vendor name | Catalog number | Dilution | Application |
| --- | --- | --- | --- | --- |
| AEP | Cell Signaling | 93627 | 1:1000 | WB |
| AEP | R&D systems | AF2058 | 1:200 | IF |
| AEP | Millipore | 6E3 | 1:1000 | WB |
| C/EBPβ | Santa Cruz | sc-7962 | 1:200 | WB |
| MAOA | abclonal | A4105 | 1:1000 | WB |
| Satb1 | Abcam | ab213363 | 1:200 | IF |
| β-actin | Sigma Aldrich | A5316 | 1:5000 | WB |
| GAPDH | ProteinTech | 60004 | 1:10000 | WB |
| COX IV | Abcam | 38197 | 1:100, 1:1000 | IF, WB |
| Sox6 | Santa Cruz | 7962 | 1:100 | WB |
| Sox6 | abclonal | A7115 | 1:1000 | WB |
| Sox6 | Invitrogen | PA581994 | 1:2000 | IF |
| ALDH1A1 | R&D systems | MAB5869 | 1:500, 1:1000 | IF, WB |
| ALDH1A1 | abcloanl | A1802 | 1:1000 | WB |
| TH | Millipore Sigma | AB947 | 1:1000 | WB |
| pS129 a-Syn | Biolegend | 825701 | 1:500 | IHC |
| a-Synuclein | abclonal | A22414 | 1:1000 | WB |
| ChaT | abcam | ab178850 | 1:500 | IF |
| His | Proteintech | 66005 | 1:5000 | WB, IF |
| GST | Proteintech | 66001 | 1:5000 | WB |
| Myc | Proteintech | 16286 | 1:5000 | WB |
| βIII tubulin | Abcam | ab18207 | 1:1000 | IF |
| EGFP | Proteintech | 66002 | 1:5000 | WB, IF |
| Bcl-2 | CST | 15071S | 1:1000 | WB |
| Sox6 N336 | abclonal | N/A | 1:1000 | WB, IF |
| Sox6 C447 | abclonal | N/A | 1:1000 | WB, IF |
| ALDH1A1 N220 | abclonal | N/A | 1:1000 | WB, IF |
| mCherry | Rockland | 600401-P16 | 1:1000 | IF |
| ProLong™ Gold Antifade Mountant with DAPI | Invitrogen™ | P36941 | -- | IF |
| Goat anti-rabbit  secondary antibody | abclonal | AS014 | 1:5000 | WB |
| alpha Synuclein Monoclonal Antibody (syn211) | Invitrogen | MA512272 | 1:1000 | WB |
| Goat anti-mouse secondary antibody | abclonal | AS003 | 1:5000 | WB |
| Rabbit anti-goat secondary antibody | Invitrogen | 31402 | 1:5000 | WB |
| Alexa FluorTM 488 Donkey anti-mouse IgG (H+L) | Invitrogen | A21202 | 1:5000 | IF |
| Alexa FluorTM 555 Donkey anti-rabbit IgG (H+L) | Invitrogen | A31572 | 1:500 | IF |
| Alexa Fluor 647 Donkey Anti-Chicken IgY (IgG) (H+L) | Jackson ImmunoResearch | 703-605-155 | 1:500 | IF |
| Alexa Fluor® 594 AffiniPure Donkey Anti-Goat IgG (H+L) | Jackson ImmunoResearch | 705-585-147 | 1:500 | IF |
| Goat Anti-Chicken IgY H&L (Alexa Fluor 594) | Abcam | ab150172 | 1:1000 | IF |
| Alexa FluorTM 647 Donkey anti-sheep IgG (H+L) | Invitrogen | A21448 | 1:500 | IF |

IF: immunofluorescence; WB: western blot.

Table S2. Human sample information

| Case | Age at onset | Sex | Primary Neuropathologic  Diagnosis | PMI  (hr) | Clinical  diagnosis | Note |
| --- | --- | --- | --- | --- | --- | --- |
| Control 1 | 65 | Male | control | 8 | lung cancer | Used in Figure 2E-I, Figure 4F-J, Figure S5C-F |
| Control 2 | 70 | Male | control | 4.5 | Cancer of pelvis | Used in Figure 2E-I, Figure 4F-J, Figure S5C-F |
| Control 3 | 59 | Male | control | 6 | lung cancer | Used in Figure 2E-I, Figure 4F-J, Figure S5C-F |
| Control 4 | 70 | Male | control | 2.5 | Control | Used in Figure 2E-I, Figure 4F-J, Figure S5C-F |
| Control 5 | 94 | Male | control | 5.5 | Control | Used in Figure 2E-I, Figure 4F-J, Figure S5C-F |
| PD 1 | 74 | Male | LBD brain stem | 4.5 | PD; possible MSA | Used in Figure 2E-I, Figure 4F-J, Figure S5C-F |
| PD 2 | 78 | Male | LBD neocortical | 10 | PD | Used in Figure 2E-I, Figure 4F-J, Figure S5C-F |
| PD 3 | 64 | Male | LBD neocortical | 13 | PD | Used in Figure 2E-I, Figure 4F-J, Figure S5C-F |
| PD 4 | 72 | Male | LBD limbic | 24 | PDD | Used in Figure 2E-I, Figure 4F-J, Figure S5C-F |
| PD 5 | 86 | Male | LBD neocortical | NA | PD; MCI | Used in Figure 2E-I, Figure 4F-J, Figure S5C-F |
| PD 6 | 72 | Male | LBD limbic | 64 | PDD | Used in Figure 2E-I, Figure 4F-J, Figure S5C-F |

PD: Parkinson’s disease; LBD: Lewy body; MSA: multiple system atrophy; MCI: mild cognitive impairment.
